# Initial learning in the brain: From rules to action

**DOI:** 10.1162/imag_a_00274

**Published:** 2024-08-20

**Authors:** Sofia Fregni, Uta Wolfensteller, Hannes Ruge

**Affiliations:** Fakultät Psychologie, Technische Universität Dresden, Dresden, Germany

**Keywords:** instruction-based learning, trial-and-error learning, MVPA

## Abstract

We used fMRI to investigate the neural changes and representational dynamics associated with different learning modes during initial learning and subsequent implementation of previously acquired stimulus-response (S-R) associations. We compared instruction-based learning (INS) and trial-and-error learning (TE) via a third observation-based learning (OBS) condition. This was yoked to the TE condition and shared features with both, the INS and TE conditions. During learning, neural changes were observed in the Frontoparietal and Default Mode Networks across learning modes, consistent with a general decrease in cognitive control demand as learning progresses. INS and TE exhibited condition-specific signal changes, which we interpreted in the context of covert motor preparation during INS, and intentional action and increased cognitive control demand during early TE trials, respectively. Multivariate pattern analysis revealed individual rule information in bilateral prefrontal, premotor, and parietal cortices across learning modes. Most regions revealed consistent representations of individual S-R rules between the learning stage and subsequent implementation stage, regardless of the learning mode. This suggests that initially formed S-R rule representations guide task performance during S-R rule implementation, irrespective of how they are acquired. Finally, within the primary motor and sensory cortices, individual S-R rules were decodable during the learning stage not only when motor responses were overtly executed, as in TE, but also in the absence of overt motor execution, as in INS. This finding substantiates previous claims of covert motor preparatory mechanisms during INS.

## 1 Introduction

Goal-directed behavior is a fundamental aspect of human experience, as it enables us to flexibly navigate the environment, and design complex plans to achieve our goals. Laboratory settings offer an avenue to study it by creating simple goals and motivating participants to attain them through more or less direct rewards, such as monetary outcomes or performance feedback. Achieving our desired outcomes in a novel environment via trial-and-error learning, which involves repeated attempts, contingent feedback, and necessary adjustments, is, however, inherently slow and error-prone. Instruction-based learning, on the other hand, refers to the unique human capacity to rapidly and efficiently learn via explicit instructions. In laboratory settings, it has been shown that humans can successfully learn newly instructed S-R links instantaneously and achieve a high degree of fluency in the space of a few practice trials (Monsell & Graham, 2021;Ruge & Wolfensteller, 2010;Wolfensteller & Ruge, 2012; cf.Baumann et al., 2023;González-García et al., 2021;Mohr et al., 2016). It follows that, at least at the behavioral level, instruction-based learning bears an indisputable advantage compared to trial-and-error learning strategies (Li et al., 2011;Monsell & Graham, 2021;Ruge, Karcz, et al., 2018). A plausible explanation to the instruction-related behavioral advantage comes from behavioral studies supporting the idea of an early, highly efficient proceduralization of instructed tasks (Oberauer, 2009,^2010^). Recently,Ruge et al. (2024)showed that instructed S-R associations can, in fact, be represented in an abstract (declarative) form, which is detached from the motor level, as well as in a format, the procedural one, that is linked to motor implementation and enables execution. In the context of trial-and-error learning, intuitively, a procedural representation is already formed during the initial rule exploration stage (Monsell & Graham, 2021;Ruge, Karcz, et al., 2018). However, the execution of wrong S-R links during exploration (Collins & Frank, 2013;Mohr et al., 2018), as well as a larger set of possible response options for each stimulus at the first attempt, might hamper rule proceduralization, which supposedly takes place less efficiently than under instruction. Support to this view comes from a recent study byMonsell and Graham (2021), in which participants engaged in a rapid learning task involving novel one-to-one S-R links that could be learned via instruction or trial-and-error. To explore the shift from declarative to procedural representation of the S-R links, the authors used between-stimulus phonological similarity as an index of declarative WM. Interestingly, phonological similarity negatively affected performance during the implementation of previously instructed S-R mappings only for a few practice trials, whereas it took three times the amount of practice trials to reach the same asymptotic performance level in the trial-and-error manipulation. This favors the hypothesis that instruction-based learning encourages an overall more efficient proceduralization during the implementation of previously instructed rules (cf.Formica et al., 2020;González-García et al., 2021;Ruge, Karcz, et al., 2018) as compared to trial-and-error learning. Interestingly, there is evidence that rule proceduralization is not only very efficient under instruction, but that it also takes place in the absence of overt motor execution and before actual rule implementation (González-García et al., 2021; see alsoMeiran et al., 2015;Palenciano et al., 2021). Specifically, numerous studies have shown that merely instructed (i.e., never overtly implemented) S-R rules can negatively impact behavioral performance when these are incongruent with the response requirements in a subsequent task (e.g.,Braem et al., 2019;Brass et al., 2009;Entel et al., 2014;Liefooghe & De Houwer, 2018;Liefooghe et al., 2012;Muhle-Karbe et al., 2017;Ruge et al., 2024;Wenke et al., 2015).Ruge and Wolfensteller (2010)andBrass et al. (2017)proposed that this early translation of novel instructions into condition-action rules might involve mental imagery and covert S-R rule implementation during the instruction phase to guide subsequent overt response execution in the implementation stage (González-García et al., 2021; cf.Palenciano et al., 2021). This is supported by neuroimaging studies that found motor and premotor activity during the instruction period, which is detached from any overt motor response implementation (Hampshire et al., 2019;Hartstra et al., 2011;Ruge & Wolfensteller, 2010). A study that investigated the hypothesis of a translation of instructed rules from the declarative to the procedural format at the neural level (Muhle-Karbe et al., 2017) compared how the same S-R instructions were represented in the brain when these had to be implemented or recalled later on. Instructions were found to be decodable in bilateral frontoparietal regions during the instruction period similarly in implementation and memorization blocks. However, during the delay period following instruction, instructions were only decodable in implementation blocks (cf.González-García et al., 2021). Furthermore, the authors compared the similarity between instruction-related patterns during the instruction and the delay period in frontoparietal regions and found greater similarity in memorization versus implementation blocks, as well as between the instruction period of implementation blocks and the delay period of memorization blocks. These findings, together, support the idea that different cognitive states might be involved during the instruction and the subsequent delay period when instructions have to be implemented later, possibly reflecting a transition from a declarative representation to a procedural one. Additionally, better instruction decoding in frontoparietal areas during the instruction period was associated with better instruction decoding in visual regions during the delay in implementation but not memorization blocks. And higher decoding accuracy in visual regions during the delay was linked to faster reaction times during rule implementation. This, in turn, supports the hypothesis that frontoparietal regions might indirectly aid later performance by tuning sensorimotor regions already during instruction encoding, and therefore support instruction proceduralization (cf.Ruge & Wolfensteller, 2010).

Following up on these earlier findings on instruction-based proceduralization, the first goal of our study was to characterize the neural representation of individual rule identities during the initial phase of learning via instruction and examine whether the early preparatory mechanisms that seems to underlie instruction-based learning, and that might be at the base of its behavioral advantage compared to trial-and-error learning, leave a signature in specific regions in the brain. Particularly, we aimed to test whether preparatory mechanisms might be visible in brain regions that are related to motor preparation and implementation already during the instruction period (i.e., without overt motor implementation), and whether these neural patterns are consistent with those supporting later rule implementation. A previous investigation byRuge et al. (2019)combined the approach to track learning-related neural changes on a trial-by-trial basis with multivariate analysis to identify brain activity patterns specific to individual S-R rule identities (e.g., “house-right index finger”; “flower-right middle finger”) during the initial implementation stage after first-time instruction. Identity-specific S-R rule representations were found in the left ventrolateral prefrontal cortex (VLPFC) as early as from the first implementation trial after instruction. The focus on implementation trials, however, raises questions about whether and how these brain representations are already built during preceding instruction trials and how this compares to different learning modes. With this in mind, we employed a task design similar toRuge et al. (2019), but novel in the way it allowed us to broaden the focus to the study of the initial phase of learning, prior to actual S-R rule implementation. Hence, in our present study, and different from a number of earlier imaging studies (Hampshire et al., 2016,^2019^;Ruge et al., 2019), we distinguished initial learning from subsequent implementation, thereby preventing the possible conflation of the two phases. This allowed us to examine how individual S-R rules might be differentially represented in the learning and implementation stages and whether these representations stay consistent from one stage to the other. This cross-stage consistency analysis was critical in providing insights particularly into whether the neural representations formed during instruction-based learning trials, which do not require response implementation, are carried forward into the implementation of the same rules and are therefore preparatory in guiding later performance.

The second goal of the present study was to investigate how the neural implementation of rules during the initial phase of learning via instruction compares to the fundamentally different trial-and-error learning. At the neural level, the literature suggests that similar brain mechanisms might support both learning types. In fact, common activation clusters supporting human value-based learning and decision-making in the brain encompass regions that have been found to increase in activity from the early implementation of previously instructed S-R rules (Ruge & Wolfensteller, 2010,^2013^), as well as during the delay following first-time instruction (Muhle-Karbe et al., 2017). These regions include frontal and parietal cortices, as well as the ventral striatum, pre-SMA, and anterior insula (Liu et al., 2011). In addition, there is evidence for similar neural dynamics underlying trial-and-error and instruction-based learning. These include a frontoparietal decrease and Default Mode Network (DMN) activity increase (e.g.,Bédard & Sanes, 2009;Hampshire et al., 2016,^2019^;Mohr et al., 2016;Ruge & Wolfensteller, 2010,^2013^), as well as an activity increase in the ventral striatum (Ruge & Wolfensteller, 2010,^2015^;Ruge et al., 2019), known for its sensitivity to various types of rewards, including positive feedback (for an overview,Daniel & Pollmann, 2014). Potential differences, however, might emerge from multivariate analysis studies, which are generally more sensitive in detecting more complex and subtle patterns than univariate analysis. Yet, in this context, instruction-based learning was never directly compared to trial-and-error learning. We therefore employed univariate and multivariate analysis to track the neural changes and representational dynamics of S-R rules while being instructed and compare them to rules that were explored via trial-and-error. To be able to compare the fundamentally different instruction-based and trial-and-error learning modes, we included a third learning condition of interest, termed observation-based learning. In this condition, learning required the mere observation of S-R rules and hence did not require actual response implementation, like in instruction-based learning. However, rules could be correct or incorrect, as indicated by post-rule feedback, requiring rule extraction, akin to trial-and-error-learning. Finding an effect specific to trial-and-error but not instruction- and observation-based learning would link the effect to motor implementation, whereas an effect common to trial-and-error and observation but not to instruction would link the effect to feedback/error processing. Finally, observing an effect common to instruction- and observation-based learning but not to trial-and-error would possibly mirror learning-related processes in the absence of overt response implementation. Our MVPA analysis covered several regions of interest (ROIs) that had previously been identified as underlying instruction-based and trial-and-error learning in the existing literature. These included the ventral and dorsal lateral prefrontal cortex (VLPFC and DLPFC;Ruge et al., 2019), the inferior and superior parietal cortices (IPC and SPC,Bode & Haynes, 2009;Cole et al., 2010;Ruge & Wolfensteller, 2010,^2013^), as well as the left motor and bilateral premotor cortices (motor and PMC,Hampshire et al., 2019;Hartstra et al., 2011;Ruge & Wolfensteller, 2010). The inclusion of regions related to motor preparation and motor implementation (as the motor and premotor cortices) in the set of ROIs is of particular relevance to our hypothesis that mere instruction trials would already involve covert motor preparation, possibly mirroring an early proceduralization of rules while being instructed. Last, this complements recent findings byAriani et al. (2022), demonstrating the utility of multivariate compared to univariate analysis in dissociating neural effects of covert motor planning from overt motor execution.

## 2 Method

The study was pre-registered on AsPredicted (https://aspredicted.org/9q9m6.pdf). Details and changes with respect to the pre-registration are in theSupplementary Material.

### 2.1 Participants

A total of 84 German native speakers participated in the experiment. Data from 4 participants were excluded due to technical errors during the experiment. The final data set included 80 participants (53 females and 27 males, mean age = 23.92, SD = 4.37, range 18–34). Participants did not have a previous history of neurological or psychiatric disorders, had normal or corrected-to-normal vision, and were right-handed. All provided written informed consent prior to the experiment start and received payment or university credits for their participation in the study. The experimental protocol was previously approved by the Ethics Committee of the Technische Universität Dresden (EK586122019).

### 2.2 Experimental material and task

**Fig. 1.**
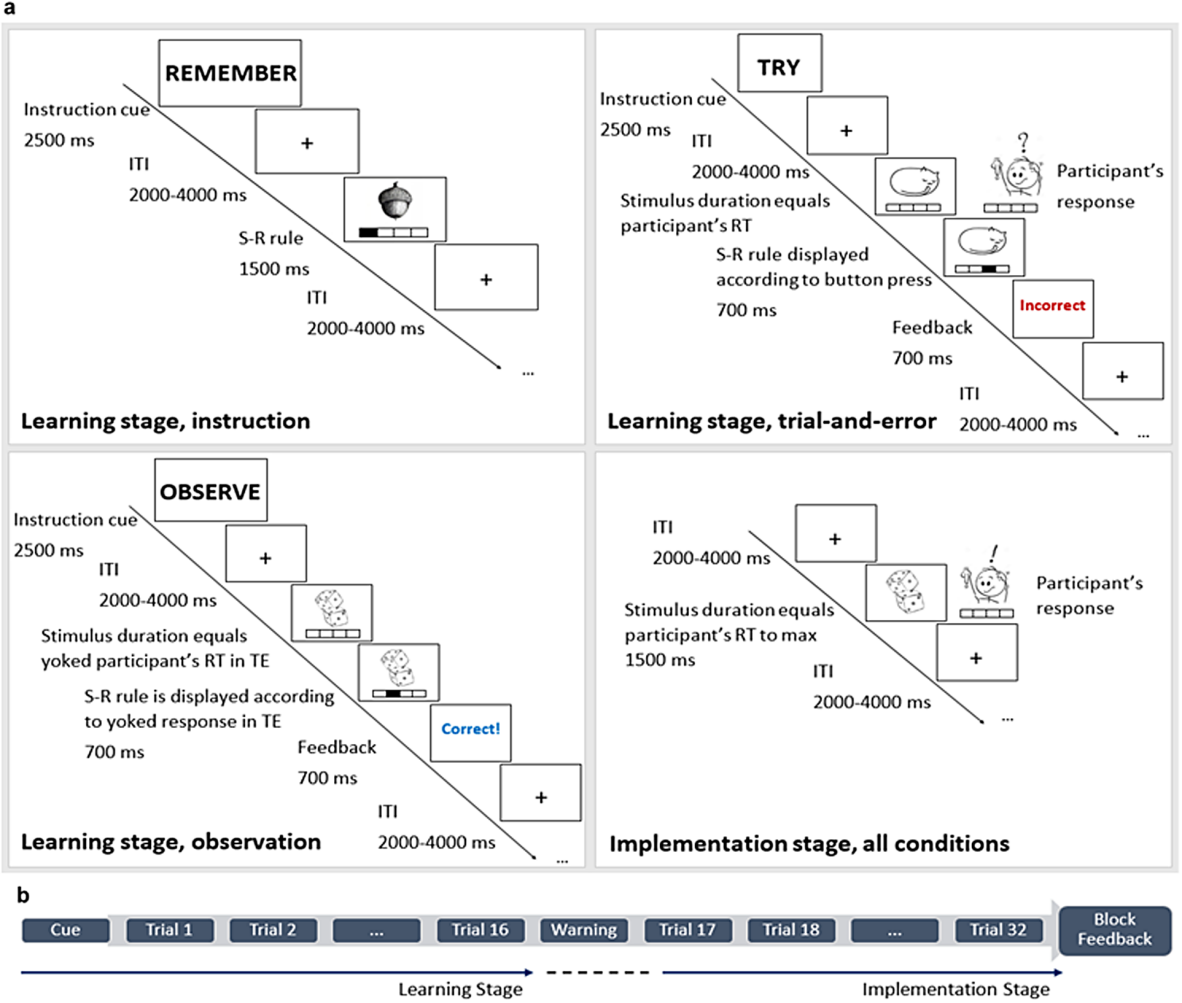
(a) The figure depicts an exemplary learning trial for each learning condition, as well as an exemplary implementation trial (same across conditions). ITI = Inter-trial interval. (b) Trial structure per each of the 24 experimental blocks. Each block comprised a total of 32 trials, 16 per each stage. Each block started with a cue, referring to the learning condition cue. After the first 16 learning trials (learning stage), a warning message reminded participants of the start of the implementation stage for the next 16 trials. At the end of each block, the corresponding block accuracy feedback was provided.

The task was a rapid learning task (Fig. 1a) that required participants to learn novel S-R links in the space of a few trials. In order to track learning processes in a trial-by-trial manner, each experimental block involved a set of 4 different novel stimuli that were coded in terms of stimulus repetitions 1 to 8. Within each block, each stimulus was repeated 4 times during the learning stage (stimulus repetitions 1 to 4), and 4 times during the implementation stage (stimulus repetitions 5 to 8). And it was linked to a unique response that participants had to learn via instruction, trial-and-error, or observation of the S-R links. Each experimental block started with a general instruction cue that informed participants about the upcoming learning condition. This was either to remember, try, or observe the S-R links (in German*Merken, Ausprobieren*or*Beobachten*) in the instruction-based, trial-and-error, and observation-based learning conditions, respectively. During the learning stage, each stimulus was presented at the center of the screen above the schematic drawing of 4 buttons. These were represented by 4 rectangles, one next to the other, one for each possible response. In the instruction-based learning condition, each stimulus was correctly paired to its response, that is, button press. The correct response that was associated with each stimulus was cued by the correspondent button on the screen that differed from the others for its black versus white color (Fig. 1a, Learning stage, instruction). In the trial-and-error learning condition, participants were asked to respond to stimuli via a button press with the index, middle, ring, or pinky finger of the right hand. At the time of the button press, the correspondent button on the screen turned black, providing participants with visual feedback about their response (Fig. 1a, Learning stage, trial-and-error). After each trial, correct or incorrect feedback was presented depending on whether the participant pressed the correct or incorrect button, respectively. In the observation-based learning condition, participants were passively attending the presentation of S-R links that could be correct or incorrect, as indicated by the following feedback (Fig. 1a, Learning stage, observation). In this sense, this learning condition resembled the trial-and-error learning condition in the measure of incorrect feedback processing. However, just like in the instruction-based learning condition, participants were not required to respond to trials during the learning stage, but only to observe what was presented on the screen. Each stimulus was presented together with the schematic drawing of 4 white buttons underneath. The identity of the observed response was cued by one of the 4 buttons on the screen that turned black after each stimulus presentation. Based on whether the observed S-R link was correct or incorrect, each trial was followed by correct or incorrect feedback, respectively.

Response onsets and response identity in the observation-based learning condition were built upon the trial-and-error learning condition as follows. Data were collected in sets of 5 participants, where the response to each trial in the trial-and-error condition was analyzed in terms of RT and accuracy (i.e., the number of correct and incorrect trials) per each individual subject. The next 5-participant set in the observation-based learning condition included trial-by-trial feedback after the observation of each S-R link that resembled the feedback that each individual participant received in the trial-and-error condition in the previous 5-participant set. This created a closed, well-controlled loop between the observation-based and trial-and-error learning conditions, as well as more naturalistic response patterns in the observation-based learning condition. Response identities and onsets in the observation-based learning condition for the first 5 participants were constructed based on previous behavioral pilot data.

The implementation stage was structured in the same way for all learning conditions and consisted of the same stimuli as in the correspondent learning stage (Fig. 1a). Participants were required to respond to trials via a button press with the index, middle, ring, or pinky finger of the right hand. No trial-by-trial feedback was presented at this stage of the experimental procedure.

The stimuli consisted of 108 black and white drawings representing various animated and inanimate objects. These were distributed across 24 experimental blocks, each consisting of 4 stimuli, plus 3 additional blocks designated for practice before the start of the experiment. The assignment of stimuli to blocks and the order of stimuli and block presentation were pseudo-randomized across participants. Importantly, stimuli sequences were built in the form of stimulus repetition chunks, where participants attended all the stimuli for the first time (stimulus repetition 1), then for the second time (stimulus repetition 2), etc… until stimulus repetition 8. Following up on earlier work (Ruge, Legler, et al., 2018), in the present study we used a refined trial sequencing scheme to eliminate bias in the planned MVPA analysis for any combination of stimulus repetitions. Different from the original version (Ruge, Karcz, et al., 2018;Ruge et al., 2019), this modified approach enabled us to compute pattern similarities during both the learning and implementation stages. Details are provided in theSupplementary Material. This allowed us to test the consistency of rule representations across the different stages of the experiment.

### 2.3 Experimental procedure

The experiment took place at the Neuroimaging Center in Dresden. Before the actual experiment, participants were familiarized with the task outside the scanner. The procedure for the practice and the real experiment were the same. The practice consisted of one block of 32 trials (4 stimuli x 8 repetitions) per each experimental condition. The 12 stimuli, 4 per condition, that were used during the practice were not re-used in the real experiment. Before the practice, participants attended a few slides with precise instructions about the task and the S-R links presentation. At this stage, participants learned to associate each finger press/response to the correspondent button that was drawn onto the screen underneath each stimulus. Following the practice, participants moved into the scanner and started the real experiment. The full experiment consisted of 24 blocks with 32 trials per block (Fig. 1b). These were administered in the form of 4 functional runs, each lasting 20 minutes and consisting of 6 blocks, with 2 blocks per condition. Block onsets within the same run were jittered with an interval of 2 or 4 seconds. Blocks were randomized across participants such that the same run for different participants always consisted of different blocks. Participants were provided with ear plugs to prevent ear damage due to the scanner noise for the whole duration of the experiment. Experimental material was projected onto the screen that is placed in the back of the scanner so that participants could see through the rear-facing mirror. Each experimental block started with a fixation cross that was presented at the center of the screen for 2 seconds. This was followed by a 2.5-seconds instruction cue that instructed participants on the upcoming learning condition. Each trial lasted a maximum of 1500 ms in all conditions or until the onset of a response in the observation-based and trial-and-error conditions. In observed trials, when a response was observed, and in trial-and-error trials, when a response was made, the button on the screen that corresponded to the button pressed in the scanner turned black for 700 ms to give visual feedback to the participants (Fig. 1a, Learning stage, observation, and Learning stage, trial-and-error, respectively). This was followed by a*Korrekt!*or*Falsch*feedback depending on whether the response was correct or incorrect, respectively. Accuracy-based feedback during learning in the trial-and-error and observation-based learning conditions lasted 700 ms and was depicted in blue for correct trials and red for incorrect trials. When no response was detected in the trial-and-error condition, a 700-ms red warning message appeared on the screen to inform participants. If participants pressed a button to respond to trials during the learning stage in the instruction- and/or observation-based learning conditions, where a response was not required, a message appeared at the center of the screen for 2.5 seconds asking them not to respond to trials and reminding them about the present learning condition. Trials were interleaved by a fixation cross that was presented at the center of the screen and jittered between 2 and 4 seconds. After 16 trials, a 2-second message was presented at the center of the screen, warning participants about the upcoming implementation stage. This consisted of another set of 16 trials, where no trial-by-trial feedback was provided. The absence of a response in implementation trials triggered a 500-ms red message warning participants that no response was detected. Like in the learning stage, the inter-trial interval was jittered between 2 and 4 seconds and trials stayed on screen until a response was made or for a maximum of 1500 ms. Each experimental block ended with individual performance-based feedback where block accuracy in the implementation stage was depicted as a percentage of correct trials in that individual block. Block-specific feedback was presented on screen for 3 seconds. At the end of the experiment, the total accuracy for the whole experiment was presented on the screen for 4 seconds. Participants were previously informed that with a total accuracy above 85% of correct trials in the whole experiment, they would have received extra money. The performance-based extra payment entailed 1 extra euro for accuracy between 85 to 89%, 2 extra euros for 90 to 94% accuracy, and 3 extra euros for overall performance above 95% accuracy. The task was coded and presented via E-Prime 3.0 software (Psychology Software Tools, Inc., 2016; RRID:SCR_009567). Stimuli sequences and experimental lists were created in Matlab (version R2020b;The MathWorks Inc., 2020; RRID:SCR_001622) and RStudio (R Core Team, 2021; RRID:SCR_000432).

### 2.4 Data acquisition

MRI data were acquired in a Siemens MAGNETOM 3T Trio Tim scanner with a 32-channel head coil. The BOLD sequence we used allowed for a good frontal resolution and minor ghosting effect (TR = 2070 ms, TE = 25 ms, flip angle = 80°, FoV = 192 mm, voxel size 3.0 × 3.0 × 3.2 mm, EPI factor = 64). Slices were acquired in descending sequential order for a total of 36 slices per volume. Each functional acquisition lasted about 25 minutes and it was followed by a GRE EPI fieldmap sequence with the same voxel size as the bold sequence in order to correct for field inhomogeneities of the bold acquisition (TR = 439 ms, TE 1 = 5.32 ms, TE 2 = 7.78 ms, flip angle = 45°, FoV = 192 mm, voxel size 3.0 × 3.0 × 3.2 mm). At the end of the 4 functional runs, a high-resolution T1-weighted anatomical image with full brain coverage was acquired for each participant (MPRAGE, TR = 1900 ms, TE = 2.26 ms, flip angle = 9°, FoV = 256 mm, voxel size = 1.0 × 1.0 × 1.0 mm).

### 2.5 Data processing

Raw MRI data were converted into BIDS 1.2.1 (Gorgolewski et al., 2016; RRID:SCR_016124) format before preprocessing. Conversion was implemented via dcm2bids (Boré et al., 2023) and Docker (Merkel, 2014).

Preprocessing was performed using*fMRIPrep*21.0.0 (Esteban, Blair, et al., 2018;Esteban, Markiewicz, et al., 2018; RRID:SCR_016216), which is based on*Nipype*1.6.1 (Gorgolewski et al., 2011,^2018^; RRID:SCR_002502).

#### 2.5.1 Preprocessing of B0 inhomogeneity mappings

A total of 4 fieldmaps per subject were used within the input BIDS structure. A*B0*nonuniformity map (or*fieldmap*) was estimated from the phase-drift map(s) measure with two consecutive GRE (gradient-recalled echo) acquisitions. The corresponding phase-map(s) were phase-unwrapped with prelude (FSL 6.0.5.1:57b01774).

#### 2.5.2 Anatomical data preprocessing

Each participant’s T1-weighted (T1w) image was corrected for intensity non-uniformity (INU) with N4BiasFieldCorrection (Tustison et al., 2010), distributed with ANTs 2.3.3 (Avants et al., 2008; RRID:SCR_004757), and used as T1w-reference throughout the workflow. The T1w-reference was then skull-stripped with a*Nipype*implementation of the antsBrainExtraction.sh workflow (from ANTs), using OASIS30ANTs as target template. Brain tissue segmentation of cerebrospinal fluid (CSF), white-matter (WM) and gray-matter (GM) was performed on the brain-extracted T1w using fast (FSL 6.0.5.1:57b01774, RRID:SCR_002823;Zhang et al., 2001). Volume-based spatial normalization to one standard space (MNI152NLin2009cAsym) was performed through nonlinear registration with antsRegistration (ANTs 2.3.3), using brain-extracted versions of both T1w reference and the T1w template. The following template was selected for spatial normalization: ICBM 152*Nonlinear Asymmetrical template version 2009c*(Fonov et al., 2009; RRID:SCR_008796, TemplateFlow ID: MNI152NLin2009cAsym).

#### 2.5.3 Functional data preprocessing

For each of the 4 BOLD runs per subject (across all tasks and sessions), the following preprocessing was performed. First, a reference volume and its skull-stripped version were generated using a custom methodology of*fMRIPrep*. Head-motion parameters with respect to the BOLD reference (transformation matrices, and six corresponding rotation and translation parameters) are estimated before any spatiotemporal filtering using mcflirt (FSL 6.0.5.1:57b01774;Jenkinson et al., 2002). The estimated*fieldmap*was then aligned with rigid-registration to the target EPI (echo-planar imaging) reference run. The field coefficients were mapped on to the reference EPI using the transform. BOLD runs were slice-time corrected to 1.01 second (0.5 of slice acquisition range 0–2.02 seconds) using 3dTshift from AFNI (Cox and Hyde, 1997; RRID:SCR_005927). The BOLD reference was then co-registered to the T1w reference using mri_coreg (FreeSurfer) followed by flirt (FSL 6.0.5.1:57b01774;Jenkinson & Smith, 2001) with the boundary-based registration (Greve & Fischl, 2009) cost-function. Co-registration was configured with six degrees of freedom. The BOLD time-series were resampled into standard space, generating a*preprocessed BOLD run in MNI152NLin2009cAsym space*. First, a reference volume and its skull-stripped version were generated using a custom methodology of*fMRIPrep*. All resamplings can be performed with a*single interpolation step*by composing all the pertinent transformations (i.e., head-motion transform matrices, susceptibility distortion correction, and co-registrations to anatomical and output spaces). Gridded (volumetric) resamplings were performed using antsApplyTransforms (ANTs), configured with Lanczos interpolation to minimize the smoothing effects of other kernels (Lanczos, 1964). Non-gridded (surface) resamplings were performed using mri_vol2surf (FreeSurfer).

Many internal operations of*fMRIPrep*use*Nilearn*0.8.1 (Abraham et al., 2014; RRID:SCR_001362), mostly within the functional processing workflow. For more details of the pipeline, see the section corresponding to workflows in*fMRIPrep*’s documentation.^*^

### 2.6 Data analysis

#### 2.6.1 Behavior

Behavioral analysis was implemented in RStudio (R Core Team, 2021; RRID:SCR_000432). Accuracy and response times (RTs) were analyzed in each condition in the implementation phase and in the trial-and-error learning stage. Data were subjected to repeated-measures ANOVAs (R package “afex”;Singmann et al., 2024) where accuracy or RT was the dependent variable as predicted by stimulus repetition for trial-and-error learning trials, and both stimulus repetition and learning condition for the analysis of implementation trials. ANOVA models included Greenhouse–Geisser sphericity correction. Each model included the random factor subject. Results with p < .05 were further investigated via post-hoc estimated marginal means tests and pairwise comparisons (R package “emmeans”;Lenth, 2024) between stimulus repetition and condition depending on the model and the significant effects. Statistics of pairwise comparisons were adjusted for the number of tests via the multivariate t-distribution approach that is available within the R package “emmeans”.

#### 2.6.2 Univariate analysis

Preprocessed functional images were processed in SPM12 software package (Statistical Parametric Mapping, RRID:SCR_007037) and its implementation in Matlab (version R2020b;The MathWorks Inc., 2020; RRID:SCR_001622). The first 3 scans for each run were discarded before starting image processing. Single-sub statistical maps were produced in the context of the General Linear Model, GLM. Correct and incorrect trials were modeled separately, excluding the very first trial in the learning phase and in the implementation phase. Trial presentation was locked to the onset of the stimulus. For both correct and incorrect trials, the original stimulus repetitions were re-coded based on accuracy (i.e., first correct/incorrect repetition, second correct/incorrect repetition, etc…). Incorrect trials were modeled for the trial-and-error and observation-based learning conditions. For the learning phase of the instruction-based learning condition, all 16 trials were considered correct. The final GLM included regressors for the first trials of both the learning and implementation stages, instruction cues, implementation stage warning start message, as well as block feedback and sustained activity. Event-related model regressors were constructed by modeling each event as a stick function convolved with the canonical Hemodynamic Response Function, HRF. The model included a high-pass filter with a 200 second cut-off.

For each subject, we created contrasts for the linear increase and decrease across stimulus repetitions in both the learning and the implementation stages, separately per each learning condition. In the context of implementation trials, we additionally implemented single-subject contrasts for the mean across repetition levels, separately for each learning condition. This was meant to test for the main effect of condition at the neural level, which was behaviorally observed uniquely in implementation trials. Before group-level statistics, single-subject contrast T-maps were smoothed with a 6 mm kernel and the SPM intra-cerebral volume mask was used to correct for voxels that lied outside of the brain. At the group level, individual statistical maps of learning trials were investigated in the context of a repeated-measures ANOVA to test the increase and decrease in brain activity during each learning condition, as well as differences between conditions. Additionally, we implemented conjunction analyses to test for brain regions that during learning commonly increased or decreased in BOLD signal across learning conditions, as expected from the previous findings (i.e., frontoparietal decrease and Default Mode Network increase), and that showed a learning condition-specific increase or decrease as compared to the other learning conditions. In the context of implementation trials, we implemented two separate group-level repeated-measures ANOVAs. One aimed to test for the repetition-wise linear increase and decrease per each learning condition and included a conjunction of contrasts to assess regions that showed a common increase/decrease across conditions. The other tested for differences between learning conditions as the mean across repetition levels and included a conjunction of contrasts to investigate differences in brain activity regarding the mean across repetition levels for each learning condition as compared to the others.

Based on the different hypotheses, we meant to test for the across-condition and condition-specific signal change, respectively, we employed two different methodological approaches in the context of the conjunction analyses for learning and implementation trials alike. To elaborate, when testing for regions that exhibited a common increase or decrease across learning conditions in learning or implementation trials, we meant to reject the null hypothesis based on a number of activations k > 2, where 2 is the number of contrasts that are included in the conjunction (linear increase per each learning condition), that is, 3 minus 1. On the other hand, when testing for brain regions where activity changed more for one condition than the others, either in a repetition-wise manner for learning trials or across-repetition in implementation trials, we used the global null hypothesis, which is based on a number of activations k = 0 (cf. congruent and incongruent contrasts inFriston et al., 2005;Nichols et al., 2005). Brain statistical maps inspection started at p(unc.) < .001. Results that are reported in this manuscript survived multiple comparison correction at the cluster level with p(FWE) < .05.

#### 2.6.3 Multivariate analysis

##### 2.6.3.1 Single trial estimation

In preparation for the identity-specific pattern similarity analysis, we estimated BOLD activity for individual trials using the Least Squares-Separate, LSS, approach (Mumford et al., 2012,^2014^). The analysis was conducted in SPM12 software package (Statistical Parametric Mapping, RRID:SCR_007037) and implemented in Matlab (version R2020b;The MathWorks Inc., 2020; RRID:SCR_001622). For each experimental trial, correct and incorrect trials alike, we ran a single-trial GLM that included the event of interest, that is, single trial, which was locked to the onset of the stimulus, and as separate regressors, all the other trials of the same condition, the trials of the other two conditions, and the instruction cue, again separately for each condition. This amounted to a total of 7 regressors per LSS model.

Event-related model regressors were constructed by modeling each event as a stick function convolved with the canonical HRF. The LSS analysis comprised a total of 768 single-trial GLMs (32 trials x 24 blocks) across 4 functional runs and included a high-pass filter at a 200 second cut-off. The first 3 scans per run were discarded before starting the analysis.

##### 2.6.3.2 Identity-specific pattern similarity

The MVPA analysis was conducted using custom-made, in-house Matlab code, which incorporated functions from the CoSMoMVPA toolbox (version 1.1.0;Oosterhof et al., 2016) and its implementation in Matlab (version R2020b;The MathWorks Inc., 2020; RRID:SCR_001622). The analysis included a whole-brain searchlight and an ROI-based MVPA. Single-trial activity T-maps were resampled from a 2 to a 3 isotropic voxel size before submitting them to both ROI-based and searchlight MVPA. This significantly reduced processing time. Importantly, different from the univariate analysis, in the MVPA we included correct and incorrect trials alike. The rationale for including incorrect trials was guided by the stimulus repetition structure, initially designed to prevent biased MVPA results (Ruge, Legler, et al., 2018). Details are provided in theSupplementary Material. Excluding error trials would have disrupted the repetition chunk setup, as described in detail inSection 2.2. In a similar vein, in the whole-brain as well as in the ROI-based MVPA, only stimulus repetitions 2 to 8 were included. The exclusion of repetition 1 was motivated by the high error rate observed at this stimulus repetition level in the trial-and-error and observation-based learning conditions. Conversely, the error rate at stimulus repetition 2 in trial-and-error and observation-based learning trials was sufficiently low not to underestimate pattern similarities due to the inclusion of a disproportionate number of incorrect trials. Therefore, we coded the learning stage as stimulus repetitions 2 to 4, and the implementation stage as repetitions 5 to 8. For each learning block, rule-identity pattern similarities were estimated as the mean differences between correlation values for the re-occurrence of the same stimulus and the occurrence of different stimuli. Correlation values were based on vectors comprising activity values for the voxels within a searchlight sphere or within a predifined ROI. Pattern similiarities obtained for each block were averaged together for each learning condition.

##### 2.6.3.3 Searchlight

First, we implemented a spherical searchlight with a 3-voxel, 9-mm radius, to produce a whole-brain map of regions where it was possible to decode rule-identities across all learning conditions and repetition levels. This was used to explore at a general level whether and where in the brain individual S-R rules could be decoded by means of all the information available we had in terms of learning conditions and stimulus repetitions (excluded stimulus repetition 1, seeSection 2.6.3). In addition, this whole-brain perspective also ensured that the more detailed pre-planned ROI-based MVPA would not miss relevant regions. At the group level, individual maps for stimulus repetitions 2 to 8, across learning conditions were submitted to one-sample t-tests and corrected for multiple comparisons at the cluster level in the context of FWE correction (p < .05).

##### 2.6.3.4 ROI-based MVPA

**Fig. 2.**
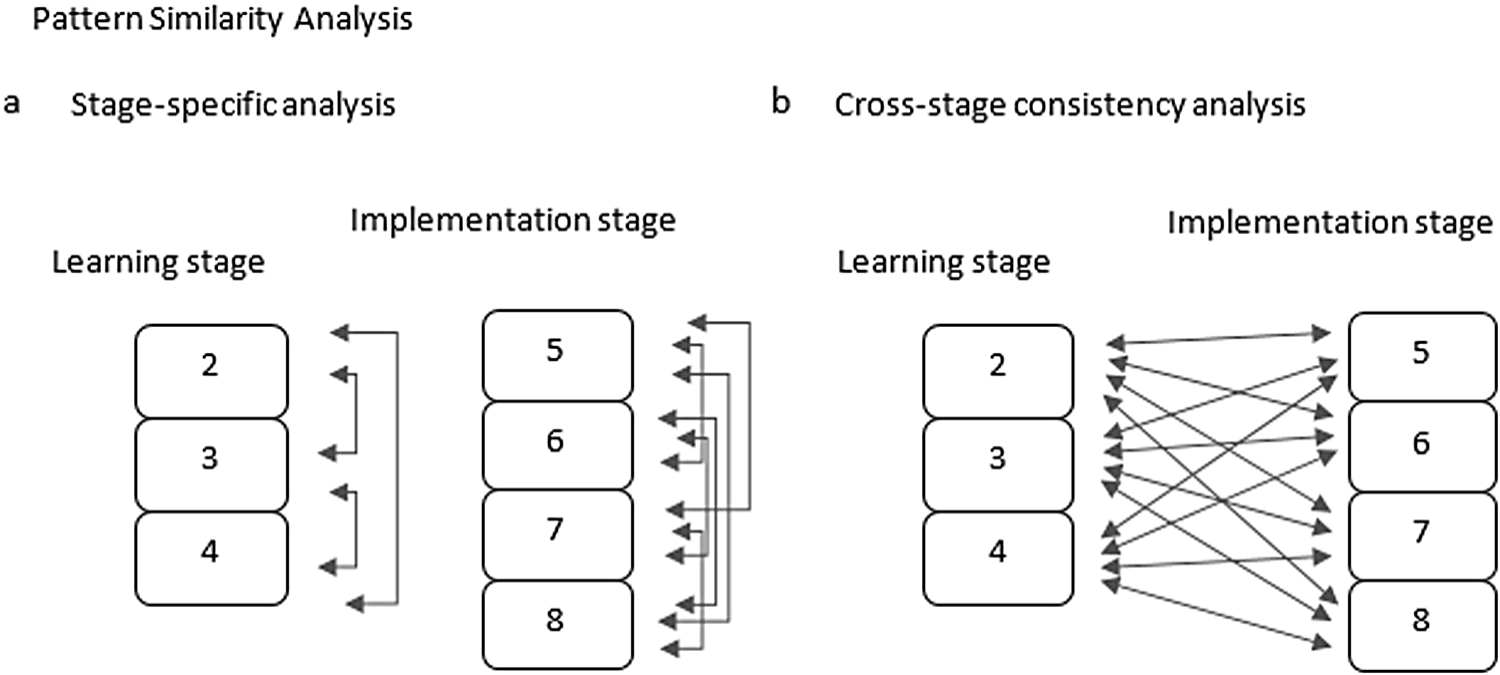
Schematic overview of pairwise comparisons in the pattern similarity analysis within the predefined ROIs. (a) Stage-specific analysis: pattern similarities between stimulus repetitions were computed within each stage before averaging and comparing one stage to the other. (b) Cross-stage consistency analysis: pattern similarity pairwise comparisons were computed between stages before averaging and comparing each mean value to the other.

The ROI-based MVPA aimed at investigating where, within predefined ROIs, it was possible to decode rule identities. Going into more detail, as compared to the whole-brain searchlight analysis, the ROI-based analysis aimed at decoding rule identities for the re-occurrence of the same stimuli within and between stages (learning vs. implementation) and separately for the three different learning conditions (instruction, trial-and-error, and observation). In this way, we were able to compare the dynamics of rule representations from the learning stage to the implementation stage and to test whether rules could be better decoded in one stage with respect to the other, or similarly in both stages. Importantly, a significant pattern similarity effect that is common to both stages but tested separately per each stage does not imply that these similarity effects are based on the same representations. To test the consistency of rule representations between stages, we computed stimulus repetition pairwise comparisons from one stage to the other.Figure 2depicts a schematic overview of how we implemented these analyses. In the*stage-specific analysis*, pattern similarity was separately determined within each stage so that each stimulus repetition was tested against each of the other stimulus repetitions within the stage of interest (Fig. 2a). Then, the mean per each separate stage was computed for each subject, and it was entered into a 4-way ANOVA with factorsstage*learning condition*region*hemisphere. Importantly, a separate 4-way repeated-measures ANOVA was computed per each ROI group (Table 1). In the*cross-stage consistency analysis*, we assessed pattern similarity across stages by aggregating stimulus repetitions 2 to 8. This allowed us to identify stable rule identity patterns from the learning stage to the subsequent implementation stage. In this context, pattern correlation between repetitions within each stage were excluded so that each stimulus repetition of the learning stage was compared to each stimulus repetition of the implementation stage (Fig. 2b). The mean of each learning-to-implementation pairwise comparison for the same stimulus repetition was then computed for each subject and compared to other stimulus repetitions learning-to-implementation pairwise comparison means in the context of a 3-way ANOVA with factorslearning condition*region*hemisphere. A separate 3-way repeated-measures ANOVA was computed per each ROI group (Table 1).

**Table 1.** List of pre-planned ROIs for the MVPA.

ROI_group	ROI	Hemisphere	Source
Prefrontal	VLPFC vs. DLPFC	L vs. R	AAL
Parietal	IPC vs. SPC	L vs. R	AAL
Premotor	vPMC/IFJ vs. dPMC	L vs. R	Searchlight across conditions and stages
Motor	Motor	L	Searchlight across conditions on implementation trials

VLPFC = Ventrolateral Prefrontal Cortex; DLPFC = Dorsolateral Prefrontal Cortex; IPC = Inferior Parietal Cortex; SPC = Superior Parietal Cortex; vPMC = Ventral Premotor Cortex; IFJ = Inferior Frontal Junction; dPMC = Dorsal Premotor Cortex; Motor = Motor Cortex.

Group-level statistics were implemented in RStudio (R Core Team, 2021; RRID:SCR_000432). The R package “afex” (Singmann et al., 2024) was used to implement the repeated-measures ANOVAs and included Greenhouse–Geisser sphericity correction. The R package “emmeans” (Lenth, 2024) was used for the post-hoc estimated marginal means tests. Reported results passed a significance threshold of p < .05 and were adjusted for the number of tests via the multivariate t-distribution approach.

##### 2.6.3.5 Regions of interest

The predefined regions of interest included ventrolateral and dorsolateral PFC as inRuge et al. (2019), as well as the superior and inferior parietal cortices in both hemispheres. Prefrontal and parietal ROIs were extracted from the AAL brain atlas (Tzourio-Mazoyer et al., 2002) that is available in the SPM12 toolbox WFU PickAtlas (Maldjian et al., 2003,^2004^). The VLPFC ROI included the Inferior Frontal Gyrus (IFG) pars triangularis and opercularis, separately in the left and right hemispheres, whereas the DLPFC included the Middle Frontal Gyrus (MFG), separately in the left and right hemispheres. In addition, we extracted ad-hoc ROIs starting from the whole-brain searchlight analysis. These were created via the SPM12 package bspmview (v. 20180918;Spunt, 2016). Specifically, we hypothesized that motor planning might play a role during learning across learning modes, even in the absence of overt motor response, like in instruction-based learning. This hypothesized role may be reflected in the brain by premotor rule decodability from the early stage of learning. Therefore, we extracted two 12-mm spherical ROIs in each hemisphere, in the ventral and dorsal premotor cortex. These were centered at different peak coordinates of the left and right premotor clusters that were found to contain S-R rule representations in the searchlight analysis that spanned all learning conditions and stimulus repetitions (right dPMC, MNI 42 -15 57; right vPMC/IFJ MNI 48 6 33; left dPMC -25 -13 63; left vPMC/IFJ MNI -43 3 42). Furthermore, based on the discovery that covert motor preparation preceding overt execution is reflected by effector-specific MVPA effects, as demonstrated byAriani et al. (2022), we aimed to investigate whether the motor cortex contralateral to response implementation in implementation trials is involved during learning even when overt motor implementation is absent, as in the case of our instruction- and observation-based learning conditions. Hence, we extracted a 12-mm spherical ROI centered at the peak activation of the left motor cluster (MNI coordinates -46 -28 63) that exhibited decodable rules in the searchlight analysis across learning conditions on implementation trials only, stimulus repetitions 5, 6, 7, and 8. Since during implementation trials all learning conditions required response implementation, we ensured that the left motor cluster containing individual S-R rule representations could not be influenced by differences between conditions, as might have occurred during the learning stage. Our rationale was that if instruction-based learning involves motor planning via covert practice as early as during the instruction period, then this should be detectable in the pattern similarity analysis within the left motor ROI during instructed trials.Table 1lists in detail the ROIs that we included in the subsequent MVPA.

## 3 Results

### 3.1 Behavior

Tables of the descriptive statistics of trial-and-error learning and implementation trials are available in theSupplementary Material.

#### 3.1.1 Trial-and-error learning trials

A repeated-measures ANOVA on accuracy in trial-and-error learning trials revealed a main effect of stimulus repetition (F[2.68,212.00] = 1,255.02, MSE = 0.00455, p < .001,${\hat{\eta }}_{p}^{2}=.941$). A post-hoc analysis showed that accuracy in trial-and-error learning trials significantly increased from one stimulus repetition to the other (all pairwise comparisons, t(79) > 7.5, p(t) < .0001).

**Fig. 3.**
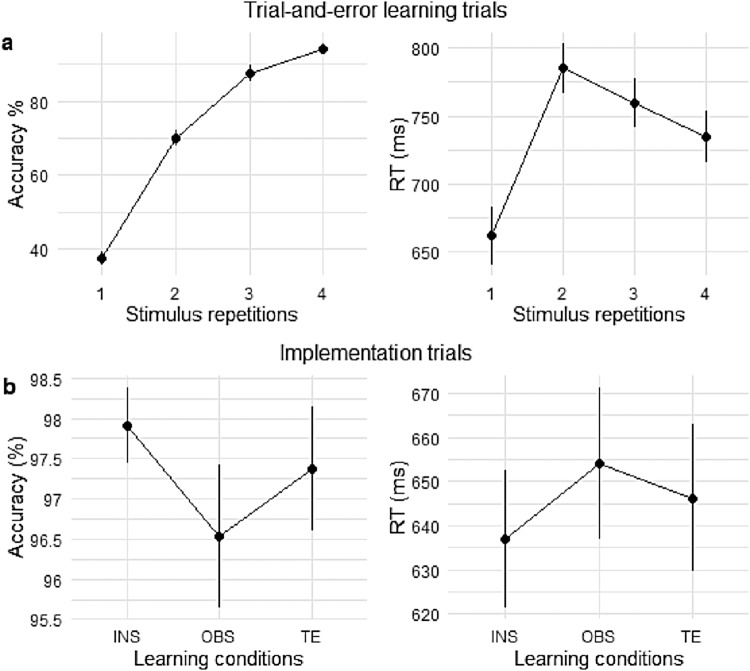
(a) Behavioral performance increase per repetition level in Trial-and-Error learning trials. 95% confidence intervals are plotted. (b) Difference between conditions in accuracy and RTs per each learning condition in implementation trials. INS = instruction-based learning; OBS = observation-based learning; TE = trial-and-error learning. 95% confidence intervals are plotted.

A repeated-measures ANOVA on RT in trial-and-error learning revealed a main effect of stimulus repetition on RTs (F[2.01,159.04] = 63.42, MSE = 5,276.48, p < .001,${\hat{\eta }}_{p}^{2}=.445$). Post-hoc, RTs showed a significant increase from stimulus repetition 1, at which point participants were guessing, to stimulus repetition 2, during which they had to retrieve the previous S-R link. From stimulus repetitions 2 to 4, RTs significantly decreased from one repetition to the other, probably reflecting task automatization. All pairwise comparisons between stimulus repetitions revealed to be significant (all comparisons, t(79) > 3.7, p(t) < .002).Figure 3ashows the effect of stimulus repetititon on accuracy and RTs in trial-and-error learning trials.

#### 3.1.2 Implementation trials

For implementation trials, we computed a repeated-measures ANOVA on accuracy using within-subject factors*stimulus repetition*(1 to 4 in implementation trials) and*learning condition*(instruction, trial-and-error, observation). There was no effect of stimulus repetition on accuracy in implementation trials (F[2.84,224.73] = 1.40, MSE = 0.00081, p = .245,${\hat{\eta }}_{p}^{2}=.017$), suggesting that learning successfully took place before the start of the implementation stage. Learning condition significantly affected accuracy during the implementation of previously learned S-R rules (main effect of learning condition, (F[1.87,147.50] = 5.89, MSE = 0.00281, p = .004,${\hat{\eta }}_{p}^{2}=.017$). There was no interaction between the factors*stimulus repetition*and*learning condition*(F[5.03,397.13] = 2.07, MSE = 0.00093, p = .068,${\hat{\eta }}_{p}^{2}=.026$). A post-hoc analysis revealed a significantly greater accuracy in previously instructed as compared to previously observed trials (t(79)=3.60,$p_{\text{MV}t\left(3\right)}=.002$). No other pairwise comparisons between learning conditions reached significance.

The 2-way repeated-measures ANOVA to test the effect of*stimulus repetition*and*learning condition*on RT during implementation trials revealed that both, stimulus repetition (F[2.20,173.75] = 5.50, MSE = 1,752.56, p = .004,${\hat{\eta }}_{p}^{2}=.065$) and learning condition (F[1.90,150.32] = 11.68, MSE = 2,137.52, p < .001,${\hat{\eta }}_{p}^{2}=.129$) significantly affected RTs. There was no interaction effect between*stimulus repetition*and*learning condition*(F[5.06, 400.04] = 1.61, MSE = 973.59, p = .156,${\hat{\eta }}_{p}^{2}=.020$). A post-hoc analysis showed that the effect of stimulus repetition was mainly guided by significantly faster RTs at the first stimulus repetition as compared to stimulus repetition 2 (t(79)=−3.50,$p_{\text{MV}t(6)}=.004$) and 3 (t(79)=−2.94,$p_{\text{MV}t(6)}=.020$). A post-hoc analysis testing for differences in RTs between learning conditions revealed a clear advantage of the instruction condition with faster RTs of previously instructed trials over both trial-and-error (t(79)=−2.85,$p_{\text{MV}t(3)}=.015$) and observation trials (t(79)=−5.03,$p_{\text{MV}t(3)}\text{​}<.001$).Figure 3bshows accuracy and RTs per learning condition in implementation trials. Tables with accuracy and RTs per stimulus repetition are provided in the supplements.

### 3.2 Univariate fMRI analysis

#### 3.2.1 Common brain mechanisms during learning and implementation of S-R rules

Group-level statistical brain maps were first investigated in the context of repeated-measures ANOVAs testing for linear signal change during learning. In particular, we were interested in:

1. common brain mechanisms underlying all learning modes and
2. signal change relative to a specific learning mode during learning.

##### 3.2.1.1 Learning stage

**Fig. 4.**
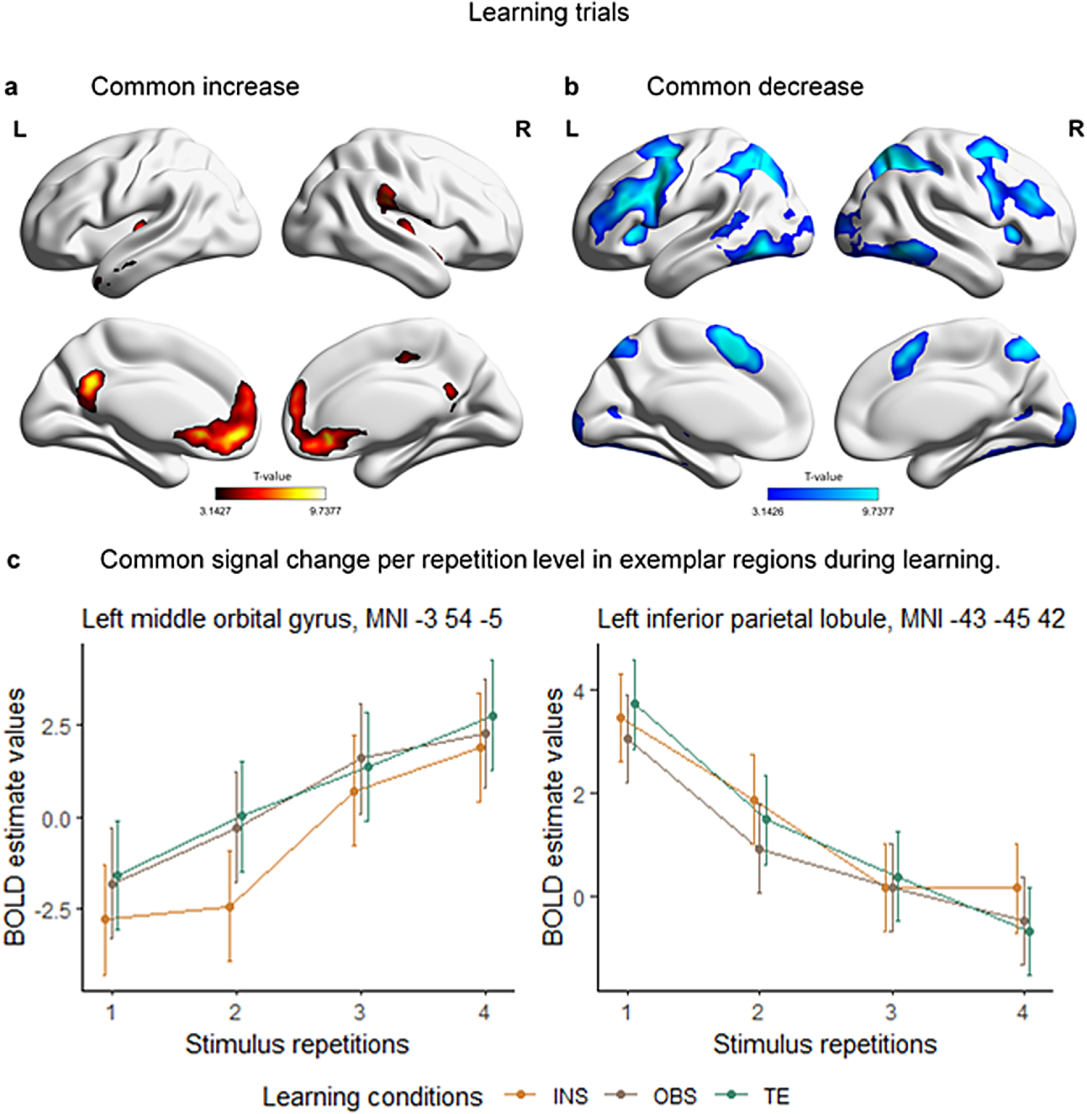
Across-condition signal change during S-R learning. (a) Medial and lateral views of T-maps exhibiting increasing activity across learning conditions. (b) Medial and lateral view of T-maps exhibiting decreasing activity across learning conditions. (c) Stimulus-repetition-wise signal increase and decrease per each learning conditions (INS = instruction, OBS = observation, TE = trial-and-error) and stimulus repetition in exemplar brain regions. 90% confidence interval are plotted. T-maps and BOLD estimate values result from the conjunction analysis (conjunction null) testing for brain regions with stimulus-repetition-wise signal increase or decrease common to all conditions during S-R learning. Results are FWE-cluster corrected (p < .05).

Figure 4shows a statistical brain map of the regions that commonly increased (Fig. 4a) and decreased (Fig. 4b) in activity during learning across learning conditions. Results were investigated in the context of a conjunction analysis that included all learning conditions’ linear increase or decrease. Brain maps were investigated at p(unc.)*< *.001 and thresholded at the cluster level at p(FWE) < .05. The BOLD linear increase during learning across all three learning conditions included frontal, temporal, and parietal regions, as well as the bilateral precuneus, middle cingulate cortex, and left posterior cingulate cortex (cf.Table 2). Most of these regions have been linked to resting-state brain activity (for an overview,Raichle, 2015). It follows that their increased activity during learning across conditions is likely to mirror brain mechanisms related to decreasing cognitive control demand. In line with our predictions and the previous literature, prefrontal, parietal, and visual regions linearly decreased in their activity during learning (cf.Table 3). We interpreted this frontoparietal control network “relaxation” as the brain mechanism accompanying the aforementioned decreasing cognitive control demand that the DMN activation might hint towards. The decrease in occipital regions, on the other hand, likely resulted from mechanisms related to stimulus habituation due to repeated exposure during learning. In fact, in particular, visuomotor learning seems to fine-tune those neurons that are responsive to the specific stimulus and response in occipital and motor regions (Makino et al., 2016, for a review on sensorimotor learning), which, in turn, would explain the decrease observed in these areas during learning.

**Table 2.** Regions that linearly increased across learning conditions during learning.

	MNI coordinates				
Region	x	y	z	Extent (voxels)	t-value (peak)
Frontal_Med_Orb_L	-3	54	-5	3957	9.74
	-3	44	-13		8.57
Olfactory_L	-1	8	-5		4.47
ACC_sub_L	-3	24	-7		7.83
ACC_sub_R	6	34	-9		8.12
ACC_pre_L	-13	48	10		5.75
Frontal_Sup_Medial_L	-3	58	24		7.09
Frontal_Sup_Medial_R	6	58	12		6.81
Frontal_Sup_2_L	-13	66	26		4.48
Supramarginal_R	52	-31	30	2123	7.75
Insula_R	42	-9	-5		6.43
Rolandic_Oper_R	54	2	2		5.80
	46	-13	20		5.46
	68	-21	16		4.26
Temporal_Sup_R	66	-5	2		4.70
	42	-31	16		3.99
Insula_L	-39	-17	6	1059	6.30
	-39	6	-17		3.39
Rolandic_Oper_L	-57	-5	8		4.13
	-45	-17	22		4.65
	-39	-33	20		3.66
Temporal_Sup_L	-41	-7	-9		5.32
	-67	-15	4		4.25
Temporal_Mid_L	-63	-9	-13		4.65
	-59	4	-25		4.97
Temporal_Pole_Mid_L	-49	14	-31		4.63
Supp_Motor_Area_R	10	-21	48	224	5.25
Cingulate_Mid_L	-7	-19	46		3.21
Cingulate_Post_L	-7	-51	32	922	9.10
Precuneus_L	-11	-57	14		5.99
Precuneus_R	6	-51	26		5.32

T = 3.14, p(unc.) < .001. Cluster-level p(FWE) < .05 (k = 224). Regions were labeled using the AAL3 atlas. Peak separation threshold = 12 mm. Regions are organized anatomically by cluster, starting from the maximal t-value.

**Table 3.** Regions that linearly decreased across learning conditions during learning.

	MNI coordinates				
Region	x	y	z	Extent (voxels)	t-value (peak)
Parietal_Inf_L	-43	-45	42	26947	14.69
	-45	-55	60		11.02
Parietal_Inf_R	50	-39	50		10.35
Angular_R	30	-53	44		13.41
SupraMarginal_L	-59	-53	32		4.54
Parietal_Sup_L	-29	-69	48		13.00
Parietal_Sup_R	18	-71	58		10.52
Precuneus_L	-9	-61	54		6.63
Temporal_Inf_L	-47	-65	-9		11.18
Temporal_Inf_R	54	-55	-11		9.79
Temporal_Mid_L	-49	-49	10		5.97
	-65	-39	-5		4.90
Lingual_L	-25	-85	-17		5.67
Fusiform_L	-37	-23	-25		4.65
Fusiform_R	36	-41	-23		6.45
Occipital_Mid_R	30	-97	10		5.69
Occipital_Sup_R	34	-73	42		12.06
Calcarine_R	10	-97	8		7.12
Calcarine_L	-11	-103	-5		6.84
Cerebellum_6_R	30	-63	-29		11.58
Cerebellum_6_L	-31	-61	-29		10.78
Cerebellum_Crus1_R	50	-77	-21		4.68
Cerebellum_Crus2_L	-7	-77	-37		6.39
Cerebellum_8_R	38	-61	-51		8.59
Cerebellum_8_L	-35	-67	-53		7.49
Cerebellum_10_L	-23	-37	-37		4.79
Supp_Motor_Area_L	-7	14	52	18017	14.03
Supp_Motor_Area_R	6	8	76		3.87
Cingulate_Mid_R	12	16	36		5.72
Precentral_L	-41	6	34		13.49
Precentral_R	48	10	32		9.48
Frontal_Mid_2_L	-27	-3	56		13.70
	-41	46	32		5.33
	-39	58	16		7.96
Frontal_Mid_2_R	44	40	32		10.39
Frontal_Sup_2_R	22	4	58		11.79
Frontal_Inf_Tri_L	-41	28	22		12.13
Frontal_Inf_Oper_R	48	14	6		3.29
Insula_L	-33	26	-1		10.80
Thal_PuA_L	-21	-31	6		5.70
Thal_VL_L	-13	-13	10		9.58
Insula_R	34	22	2	369	9.12
Thal_VL_R	12	-13	8	1336	7.71
Thal_VPL_R	26	-25	-1		4.72
Putamen_R	18	10	4		5.08
Calcarine_R	20	-71	8	462	5.65
Calcarine_L	-11	-71	10		4.76
Vermis_3	-1	-47	-23	229	4.98

T = 3.14, p(unc.)*< .*001. Cluster-level p(FWE) < .05 (k = 229). Regions labeled using the AAL3 atlas. Peak separation threshold = 20 mm. Regions are organized anatomically by cluster, starting from the maximal t-value.

Figure 4cshows two exemplar regions for the stimulus-repetition-wise linear increase and decrease in the left middle orbital gyrus, MNI -3 54 -5, and in the left inferior parietal lobule, MNI -43 -45 42, respectively.

##### 3.2.1.2 Implementation stage

Linear contrasts for the stimulus-repetition-wise signal increase and decrease per each learning condition were included in two different conjunction analyses testing brain regions that linearly increased and decreased, respectively, across learning conditions during the implementation of previously learned S-R rules.

**Fig. 5.**
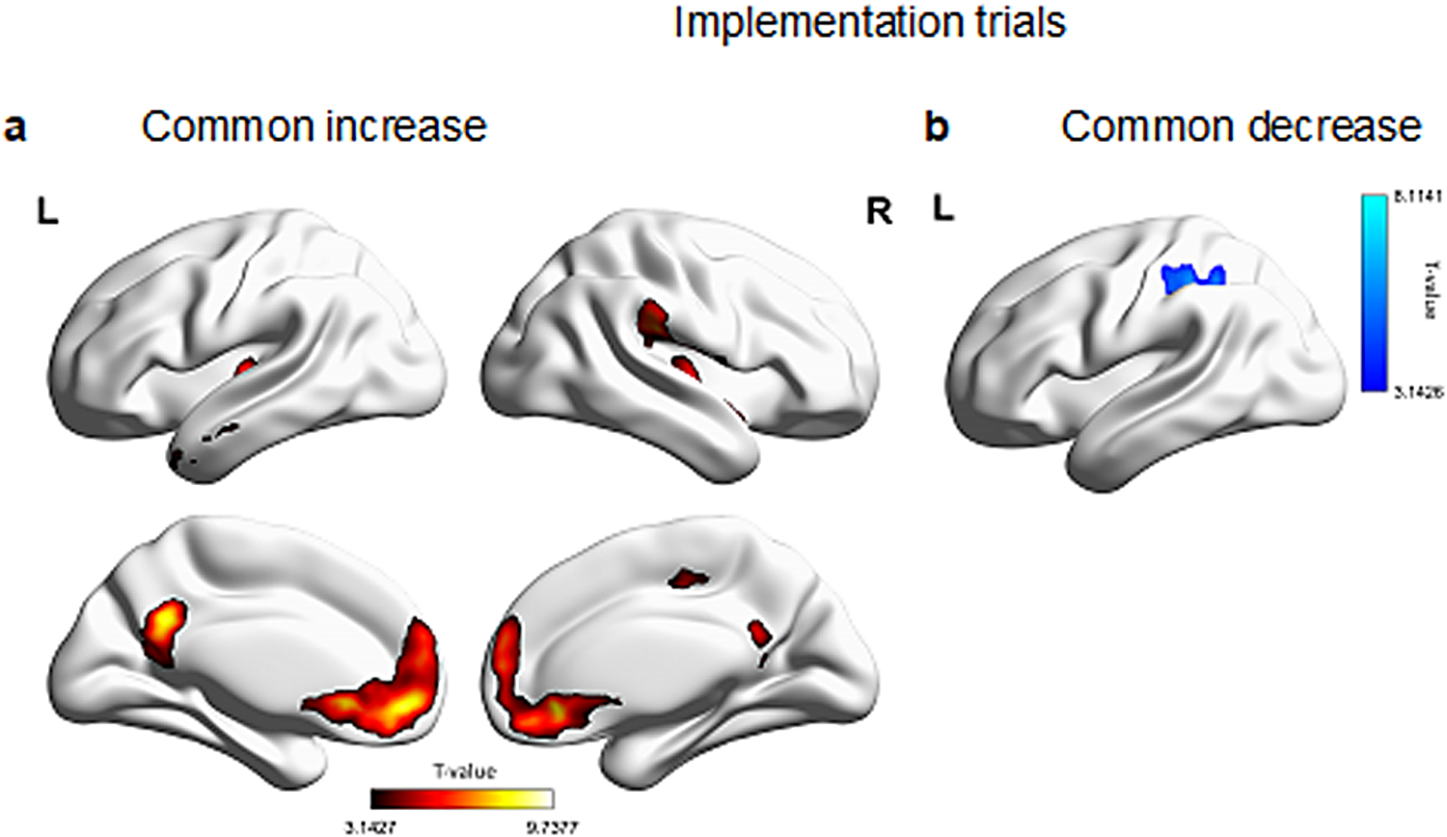
T-maps of the signal change across learning conditions in implementation trials. (a) Medial and lateral view of brain regions exhibiting increasing activity across learning conditions. (b) Lateral view of the left hemisphere (no suprathresholds cortical activations in the right hemisphere) with brain regions exhibiting decreasing activity across learning conditions. Statistical maps result from a conjunction analysis (conjunction null) testing for brain regions with stimulus-repetition-wise signal increase or decrease common to all conditions during S-R implementation. Results are FWE-cluster corrected (p < .05).

Statistical maps were investigated at p(unc.)*< *.001 and corrected for multiple comparisons at the cluster level in the context of FWE-correction (p < .05).Figure 5shows the statistical map of brain regions that linearly increased (5a) and decreased (5b) in activity during implementation trials across learning conditions. A linear increase was found in a bilateral occipital cluster, including the cuneus, motor regions in both hemispheres, as well as areas belonging to the DMN, like the angular gyrus, posterior cingulate, frontal and temporal cortex (for a complete list of activations, refer toTable 4). Brain regions in the left inferior parietal lobule, postcentral gyrus (Fig. 5b), thalamus, and putamen linearly decreased in activity during S-R implementation trials, together with a right hemisphere cluster in the cerebellum (Table 5), possibly mirroring visual- and somatomotor adaptation as sensorimotor learning proceeds (e.g.,Floyer-Lea & Matthews, 2004;Makino et al., 2016for a review).

**Table 4.** Regions that linearly increased across learning conditions during implementation trials.

	MNI coordinates				
Region	x	y	z	Extent (voxels)	t-value (peak)
Cuneus_L	-7	-103	14	7483	9.98
	-9	-91	36		7.24
Cuneus_R	12	-97	20		8.78
	12	-91	40		5.63
Occipital_Mid_L	-23	-97	12		7.36
Occipital_Mid_R	38	-87	16		4.98
	30	-81	28		4.58
Occipital_Inf_R	32	-93	-11		3.88
Lingual_R	18	-85	-11		5.68
	8	-79	-1		4.84
	24	-63	-9		3.42
Calcarine_L	-7	-91	-13		5.32
	-7	-61	12		4.46
Calcarine_R	10	-99	-5		3.22
Fusiform_L	-21	-85	-9		5.28
Fusiform_R	34	-73	-15		3.85
Cingulate_Mid_L	-15	-51	34		5.67
Cingulate_Post_R	8	-53	30		4.18
Angular_R	44	-63	28		4.15
Parietal_Sup_L	-21	-79	44		3.76
Temporal_Mid_R	48	-69	4		5.13
Temporal_Sup_R	66	-5	-11	193	3.44
Temporal_Mid_R	64	-1	-27		4.49
Temporal_Mid_L	-63	-7	-19	390	5.30
Temporal_Inf_L	-55	-1	-37		4.08
Temporal_Mid_L	-53	-67	22	290	4.94
Occipital_Mid_L	-41	-77	32		4.53
Precentral_R	50	-11	48	1063	6.51
	34	-27	54		4.90
Postcentral_R	60	-1	18		3.45
Postcentral_L	-47	-15	36	431	5.38
	-59	-7	22		4.39
Frontal_Sup_Medial_L	-3	54	2	6334	7.07
	2	56	26		6.93
Frontal_Sup_2_L	-23	32	44		5.60
	-15	56	32		5.29
Frontal_Sup_2_R	16	64	18		5.28
ACC_sub_L	-7	34	-7		6.27
ACC_pre_L	-5	38	18		3.67
N_Acc_L	-7	16	-5		5.26
OFClat_L	-47	26	-17	287	5.42

T = 3.14, p(unc.)*< .*001. Cluster-level p(FWE) < .05 (k = 193). Regions labeled using the AAL3 atlas. Peak separation threshold = 15 mm. Regions are organized anatomically by cluster, starting from the maximal t-value.

**Table 5.** Regions that commonly decreased across learning conditions during implementation trials.

	MNI coordinates				
Region	x	y	z	Extent (voxels)	t-value (peak)
Postcentral_L	-45	-35	50	1170	8.11
Vermis_4_5	-1	-51	-21	1389	6.98
Vermis_8	-1	-63	-35		6.36
Cerebellum_6_R	26	-53	-25		5.95
Cerebellum_8_R	18	-61	-47		5.11
Thal_VL_L	-19	-17	10	182	4.28
Putamen_L	-25	2	12		4.21

T = 3.14, p(unc.)*< .*001. Cluster-level p(FWE) < .05 (k = 182). Regions labeled using the AAL3 atlas. Peak separation threshold = 15 mm. Regions are organized anatomically by cluster, starting from the maximal t-value.

#### 3.2.2 Learning condition-specific signal change

##### 3.2.2.1 Learning stage

**Fig. 6.**
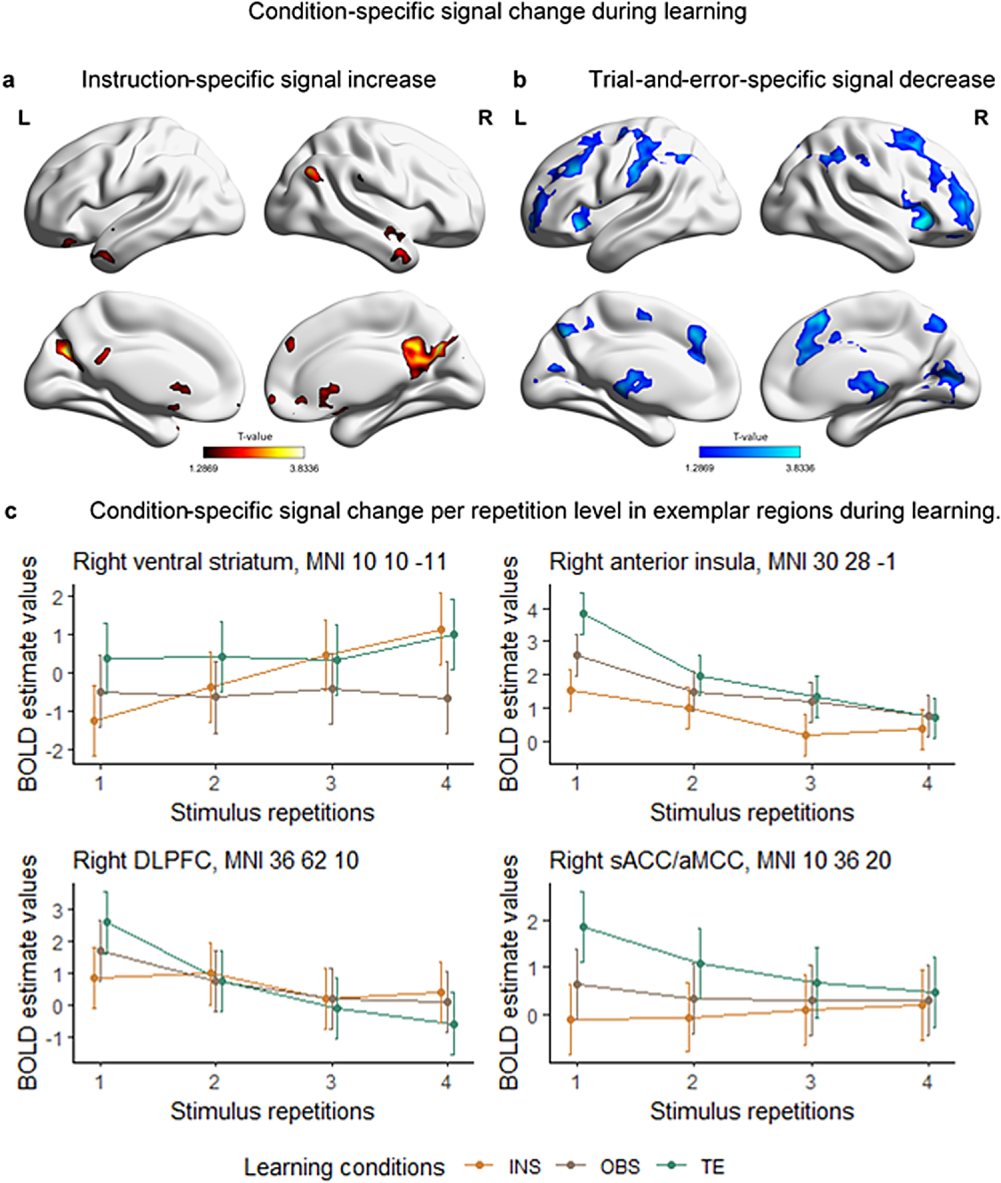
Condition-specific signal change during S-R learning. (a) Coronal and sagittal view of the brain for instruction-specific linear increase as compared to trial-and-error and observed trials. (b) Coronal and sagittal view of the brain for trial-and-error-specific linear decrease as compared to instructed and observed trials. (c) Exemplar regions for condition-specific signal change. INS = instruction, OBS = observation, TE = trial-and-error, DLPFC = Dorso-Lateral Prefrontal Cortex. T-maps (a, b) and MNI coordinates (c) result from the conjunction analyses (global null, FWE-cluster corrected, p < .05) that tested for condition-specific linear signal change during learning. BOLD estimate values come from repeated-measure ANOVA testing for the interactionlearning condition*stimulus repetition. 90% confidence interval are plotted.

In order to find condition-specific brain activity during the learning stage, we performed conjunction analyses in the context of the repeated-measures ANOVA testing for a linear increase and decrease during learning. We were particularly interested in finding brain areas that linearly decreased or increased in activity significantly more in one learning condition than in the others. After p(FWE) cluster correction, clusters of regions only survived in the conjunction testing for regions that showed an increase specific to the instruction-based learning condition and a decrease specific to the trial-and-error learning condition (Fig. 6).

##### 3.2.2.2 Instruction-based learning-related signal change

Frontal, parietal and temporal areas commonly associated with the DMN showed a greater BOLD signal increase during instructed trials as compared to observed and trial-and-error trials (Fig. 6a;Table 6). Additionally, regions in the basal ganglia showed a similar signal change pattern.Figure 6cshows the signal change in the right ventral striatum (i.e., Nucleus Accumbens, MNI 10 10 -11), as an exemplar region for the instruction-specific increase during learning.

**Table 6.** Regions with instruction-specific signal linear increase during learning.

	MNI coordinates				
Region	x	y	z	Extent (voxels)	t-value (peak)
Precuneus_R	10	-69	34	2427	3.83
Precuneus_L	-17	-67	30		3.80
Cingulate_Mid_R	10	-51	32		3.23
Cuneus_R	6	-85	26		1.43
Calcarine_R	4	-55	10		2.53
N_Acc_R	10	10	-11	2509	3.43
N_Acc_L	-11	12	-11		2.24
Caudate_R	6	20	-1		2.83
Caudate_L	-5	12	2		2.48
OFCpost_R	24	20	-17		2.97
OFCant_L	-21	30	-19		2.71
Frontal_Med_Orb_R	6	38	-9		2.39
Rectus_L	-9	22	-21		2.00
Rectus_R	14	30	-21		2.77
Temporal_Pole_Mid_R	48	18	-31		2.16
Temporal_Mid_R	56	2	-29		2.36
Temporal_Sup_R	58	-7	-13		2.31
Temporal_Mid_L	-63	-7	-17	137	2.90
Temporal_Mid_L	-51	2	-31	269	2.84
Frontal_Med_Orb_L	-1	58	-9	324	2.74
Frontal_Med_Orb_R	8	68	-9		1.79
SupraMarginal_R	68	-29	34	311	2.60

T = 1.29, p(unc.)*< .*001. Cluster-level p(FWE) < .05 (k = 137). Regions labeled using the AAL3 atlas. Peak separation threshold = 12 mm. Regions are organized anatomically by cluster, starting from the maximal t-value.

##### 3.2.2.3 Trial-and-error learning-related signal change

Frontal, prefrontal, and parietal regions, as well as bilateral clusters in the cerebellum linearly decreased in their activity more during trial-and-error learning than observed and instructed learning trials (Fig. 6b;Table 7).Figure 6cshows the signal change in the right anterior insula (AI, MNI coordinates 30 28 -1), right dorsolateral prefrontal cortex (DLPFC, MNI coordinates 36 62 10), and right supracallosal anterior cingulate cortex/anterior mid-cingulate cortex (sACC/aMCC, MNI coordinates 10 36 20), as exemplar regions for the trial-and-error-specific decrease as compared to instructed and observed trials.

**Table 7.** Regions with trial-and-error-specific signal decrease during learning.

	MNI coordinates				
Region	x	y	z	Extent (voxels)	t-value (peak)
Supp_Motor_Area_R	6	20	54	13249	3.98
Frontal_Sup_Medial_L	-3	32	40		3.82
ACC_sup_L	-13	36	22		2.69
ACC_sup_R	10	36	20		3.06
Cingulate_Mid_R	10	6	38		2.54
Frontal_Sup_2_R	36	62	10		3.85
Frontal_Sup_2_L	-25	66	22		1.79
OFCant_R	24	46	-13		3.46
Frontal_Mid_2_L	-31	56	-9		3.56
	-45	24	36		3.38
	-31	6	54		3.27
Frontal_Mid_2_R	42	36	36		3.01
	30	10	50		3.46
Frontal_Inf_Tri_R	42	18	22		1.82
Precentral_L	-31	-13	64		2.84
Precentral_R	62	12	30		1.64
Postcentral_L	-55	-21	34		3.61
Parietal_Inf_L	-47	-59	50		3.23
Supp_Motor_Area_L	-7	-7	54	128	2.64
Insula_R	30	28	-1	9732	4.78
Insula_L	-31	22	-7		4.18
Frontal_Inf_Oper_R	52	18	6		2.87
Frontal_Inf_Oper_L	-49	6	6		1.73
Putamen_L	-27	-3	-5		2.46
Putamen_R	30	-3	4		2.28
Caudate_L	-11	4	8		2.76
Caudate_R	12	10	16		3.23
Thal_PuM_R	4	-27	-1		3.62
Thal_PuI_L	-19	-25	12		2.26
Thal_PuI_R	16	-21	16		2.06
Cuneus_L	-7	-77	18		2.63
Lingual_R	6	-77	-9		2.77
Calcarine_L	4	-97	-3		2.05
Calcarine_R	14	-81	12		3.00
Cerebellum_Crus1_L	-11	-85	-23		2.74
Cerebellum_Crus1_R	18	-95	-21		2.65
Cerebellum_4_5_R	18	-55	-17		3.11
Cerebellum_6_L	-29	-75	-21		1.58
SupraMarginal_R	38	-29	36	1691	2.50
Angular_R	38	-61	38		2.43
Parietal_Inf_R	58	-45	48		3.31
Postcentral_R	42	-41	60		1.49
Parietal_Sup_R	30	-71	58		1.43
Precuneus_L	-7	-69	42	1207	3.57
Precuneus_R	20	-67	40		2.31

T = 1.29, p(unc.)*< .*001. Cluster-level p(FWE) < .05 (k = 128). Regions labeled using the AAL3 atlas. Peak separation threshold = 20 mm. Regions are organized anatomically by cluster, starting from the maximal t-value.

##### 3.2.2.4 Implementation stage

Given the significant behavioral differences between conditions but not repetition levels in implementation trials, we computed group-level brain maps for the mean signal change across stimulus repetitions during implementation trials, separately for each condition. Across-repetition contrasts as the mean signal change per each learning condition were included in three conjunction analyses, each testing for signal change that was specific to one versus the other conditions. However, no voxel survived correction, not even at p(unc.)*< *.001, in any of the conjunctions of contrasts, suggesting no main effect of learning condition at the brain level in implementation trials.

### 3.3 Mvpa

#### 3.3.1 Searchlight

**Fig. 7.**
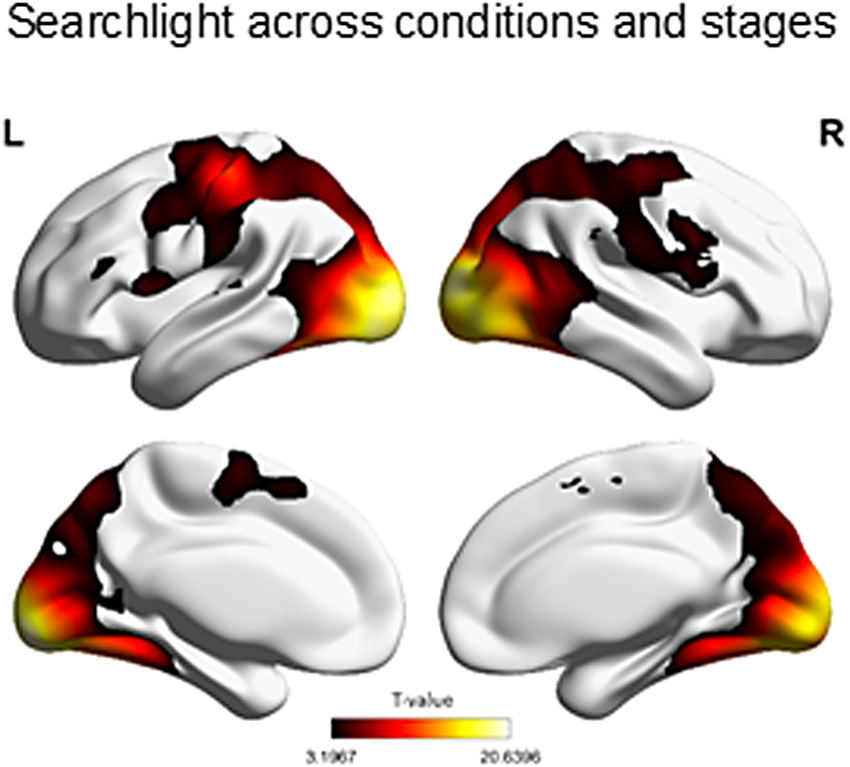
Whole-brain searchlight across learning conditions and repetition levels (stimulus repetitions 2 to 8). Statistical map was corrected at the cluster level in the context of FWE correction (p < .05).

In order to find a whole-brain map to test whether and where individual S-R rules could be decoded in the brain in general and with the highest statistical power, we implemented a whole-brain spherical searchlight with a 3-voxel (9 mm) radius that included all learning conditions and repetition levels (excluding stimulus repetition 1). After cluster correction for multiple comparisons at p(FWE) < .05, the searchlight identified pattern similarity effect in extended bilateral clusters spanning occipital, prefrontal and parietal cortices (Fig. 7;Table 8). Higher-order regions like the prefrontal and parietal cortex likely reflect S-R rule representations, as opposed to occipital and motor cortices, which mirror stimulus and response identities, respectively (Ruge et al., 2019, Experiment 2).

**Table 8.** Whole-brain map of decodable S-R rules across conditions and stages.

	MNI coordinates				
Region	x	y	z	Extent (voxels)	t-value (peak)
Occipital_Inf_L	-16	-94	-10	19238	20.64
Occipital_Inf_R	39	-85	-4		17.71
Occipital_Sup_R	27	-97	12		19.18
Occipital_Mid_L	-28	-94	6		18.69
Fusiform_L	-40	-58	-16		14.09
Lingual_R	21	-88	-7		17.37
Temporal_Mid_L	-67	-49	9		3.58
Postcentral_L	-43	-25	54		11.52
Precentral_L	-25	-13	63		5.89
	-43	3	39		5.28
Precentral_R	42	-16	57		5.60
	48	6	30		5.08
	57	6	18		4.43
Supp_Motor_Area_L	-7	-4	63		4.63
Supp_Motor_Area_R	3	6	57		4.50
Paracentral_Lobule_L	-4	-10	81		3.66
Rolandic_Oper_R	42	-16	21		3.54
Frontal_Inf_Tri_L	-49	30	18		4.10
Frontal_Inf_Oper_L	-58	6	9		6.51
Frontal_Sup_2_R	27	-1	54		4.82
Insula_R	36	-7	21		3.60
Precuneus_L	-1	-70	54		6.01
Parietal_Sup_L	-25	-67	51		6.60
Parietal_Sup_R	30	-58	57		5.68
Parietal_Inf_R	39	-40	51		5.28
SupraMarginal_L	-61	-25	24		4.43
SupraMarginal_R	54	-37	33		4.17

T = 3.20, p(unc.)*< .*001. Cluster-level p(FWE) < .05 (k = 19238). Regions labeled using the AAL3 atlas. Peak separation threshold = 12 mm. Regions are organized anatomically by cluster, starting from the maximal t-value.

#### 3.3.2 ROIs

##### 3.3.2.1 VLPFC and DLPFC

**Fig. 8.**
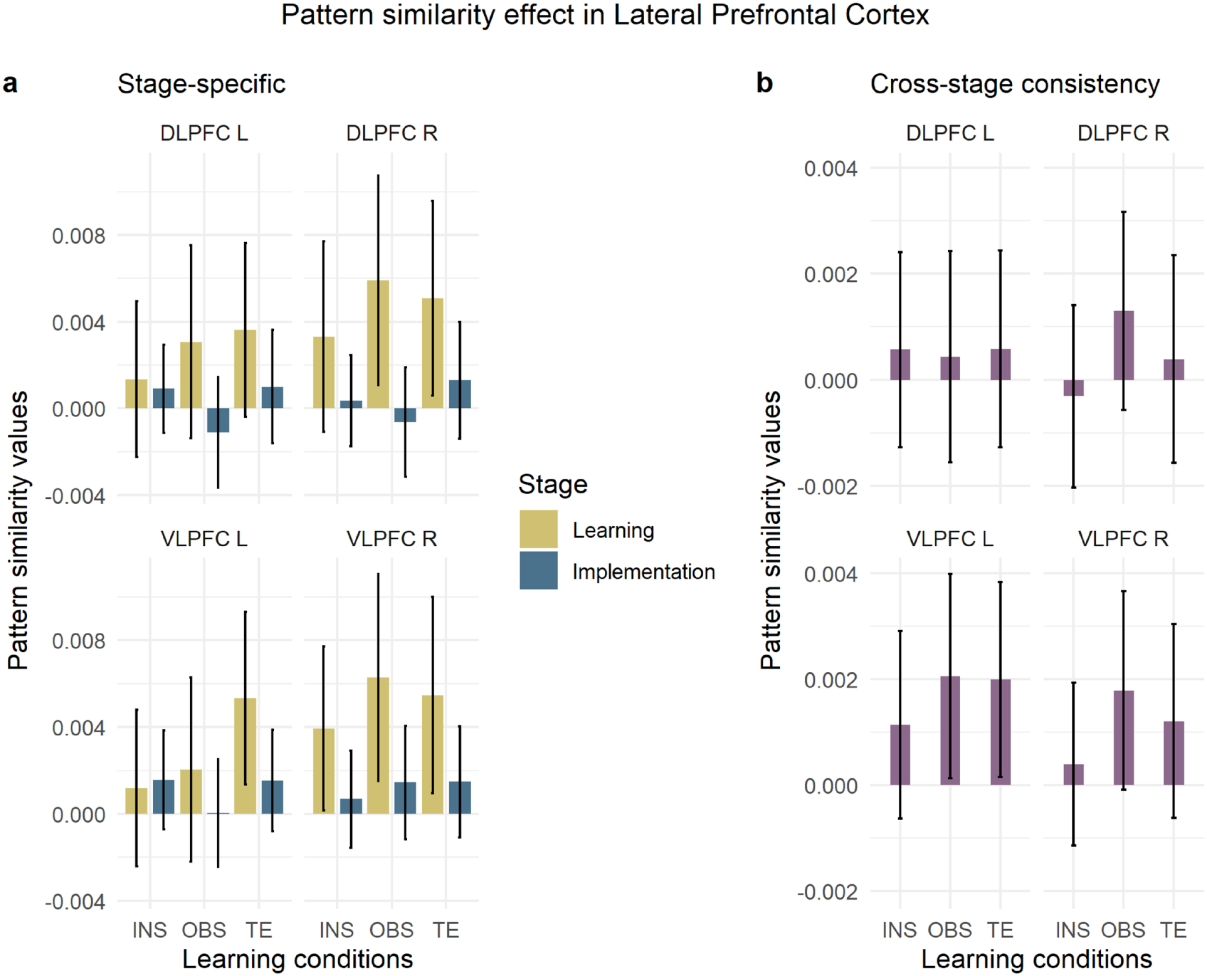
Pattern similarity effect in LPFC. 95% Confidence intervals are plotted. (a) Stage-specific pattern similarity analysis per each condition and stage. (b) Cross-stage consistency pattern similarity analysis per each condition. INS = instruction, OBS = observation, TE = trial-and-error. VLPFC = ventrolateral prefrontal cortex, DLPFC = dorsolateral prefrontal cortex.

In the stage-specific analysis, where pattern similarities were determined separately within each stage, the repeated-measures ANOVA with within-subject factorsstage*condition*region*hemisphererevealed an overall significant pattern similarity effect (Intercept, F[1,79] = 11.71, MSE = 0.00087, p < .001,${\hat{\eta }}_{p}^{2}=.129$). Pattern similarity values significantly differed between stages (main effect of stage, F[1,79] = 6.02, MSE = 0.00080, p = .016,${\hat{\eta }}_{p}^{2}=.071$) and hemispheres (main effect of hemisphere, F[1,79] = 9.65, MSE = 0.00007, p = .003,${\hat{\eta }}_{p}^{2}=.109$), with learning > implementation (t(79)=2.45,p=.016) and right > left (t(79)=−3.11,p=.003), respectively. Importantly, a pattern similarity effect significantly different from 0 was revealed only in the learning stage in both VLPFC and DLPFC (one-sample t-test, learning stage: VLPFC,t(79)=3.41,$p_{\text{MV}t(4)}=.004$, DLPFCt(79)=3.04,$p_{\text{MV}t(4)}=.011$; implementation stage: VLPFCt(79)=1.62,$p_{\text{MV}t(4)}=.303$, DLPFC,t(79)=0.41,$p_{\text{MV}t(4)}=.978$). In addition, stage and hemisphere significantly interacted (F[1,79] = 4.87, MSE = 0.00011, p = .030,${\hat{\eta }}_{p}^{2}=.058$), showing a significant difference between stages with learning > implementation particularly in the right hemisphere (t(79)=2.89,p=.005;Fig. S3ain the Supplementary Material), but not in the left (t(79)=1.64,p=.104). There was no significant effect of condition (F[1.96,154.89] = 0.46, MSE = 0.00077, p = .631,${\hat{\eta }}_{p}^{2}=.006$) or region (F[1,79] = 1.45, MSE = 0.00011, p = .232,${\hat{\eta }}_{p}^{2}=.018$), suggesting that rules were represented similarly across conditions in the VLPFC and DLPFC.Figure 8ashows the MVPA effect for each condition and region X hemisphere combination during learning and implementation, separately.

When inspecting how consistent S-R rule representations were from the learning to the implementation stage, the repeated-measures ANOVA with within-subject factorscondition*region*hemisphererevealed a marginal significant pattern similarity effect (Intercept, F[1,79] = 3.96, MSE = 0.00022, p = .050,${\hat{\eta }}_{p}^{2}=.048$). The cross-stage pattern analysis (Fig. 8b) showed a main effect of region (F[1,79] = 8.65, MSE = 0.00002, p = .004,${\hat{\eta }}_{p}^{2}=.099$) with significantly stronger rule identity patterns in VLPFC than DLPFC (t(79)=−2.94,p=.004;Fig. S3bin the Supplementary Material). The absence of an interaction effect between the factors region, hemisphere, and condition (F[2.00,157.62] = 0.86, MSE = 0.00001, p = .427,${\hat{\eta }}_{p}^{2}=.011$) suggests that S-R rules were represented similarly in the VLPFC across learning modes and hemispheres. Neither condition (F[1.99,157.19] = 0.36, MSE = 0.00021, p = .700,${\hat{\eta }}_{p}^{2}=.004$) nor hemisphere (F[1,79] = 1.03, MSE = 0.00003, p = .313,${\hat{\eta }}_{p}^{2}=.013$) showed significant effects.

We speculated on the cross-stage consistency analysis results given that the stage-specific pattern analysis revealed LPFC MVPA effects in learning but not implementation trials. We reasoned that the cross-stage consistency pattern analysis could be more sensitive than the stage-specific pattern analysis in picking up subtle but consistent patterns of activity in the implementation stage. The power of the cross-stage analysis in detecting consistency between representations, even those with subtler effects, is likely driven by the inclusion of a greater number of pairwise correlations spanning both stages (Fig. 2).

##### 3.3.2.2 IPC and SPC

**Fig. 9.**
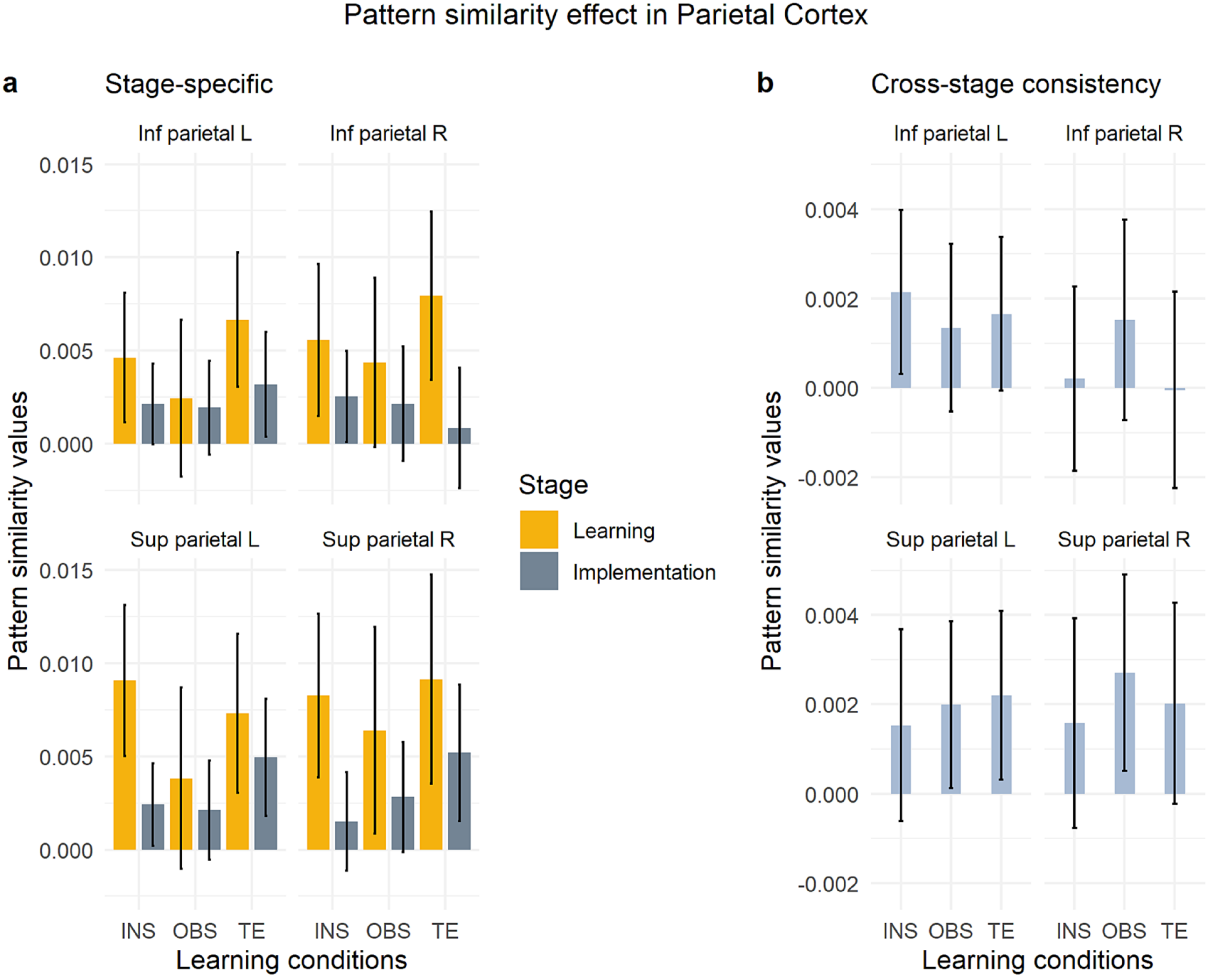
Pattern similarity effect in the inferior and superior parietal cortex. 95% Confidence intervals are plotted. (a) Stage-specific pattern similarity analysis per each condition and stage. (b) Cross-stage consistency pattern similarity analysis per each condition. INS = instruction, OBS = observation, TE = trial-and-error. Inf parietal = Inferior parietal cortex, Sup parietal = Superior parietal cortex.

In the stage-specific MVPA within the IPC and SPC ROIs, the repeated-measures ANOVA with within-subject factorsstage*condition*region*hemisphererevealed an overall significant pattern similarity significant effect (Intercept, F[1,79] = 43.27, MSE = 0.00089, p < .001,${\hat{\eta }}_{p}^{2}=.354$). There was a significant impact of stage (main effect of stage, F[1,79] = 6.93, MSE = 0.00092, p = .010,${\hat{\eta }}_{p}^{2}=.081$) and region (main effect of region, F[1,79] = 8.50, MSE = 0.00014, p = .005,${\hat{\eta }}_{p}^{2}=.097$) on S-R rule decodability, with learning > implementation (t(79)=2.63,p=.010) and SPC > IPC (t(79)=−2.92,p=.005), as depicted inFigure S4ain the Supplementary Material. In addition, stage and region significantly interacted with learning condition (3-way interactionstage*region*condition, F[1.83,144.29] = 3.53, MSE = 0.00011, p = .036,${\hat{\eta }}_{p}^{2}=.043$), with significantly stronger pattern similarity values in the learning versus implementation stage in the SPC during instructed trials (t(79)=2.79,p=.007) and in the IPC during trial-and-error trials (t(79)=2.35,p=.021). No main effect of hemisphere (F[1,79] = 1.18, MSE = 0.00010, p = .281,${\hat{\eta }}_{p}^{2}=.015$) nor condition (F[1.77,139.77] = 1.23, MSE = 0.00085, p = .293,${\hat{\eta }}_{p}^{2}=.015$) reached significance.Figure 9ashows the MVPA effect for each condition and region X hemisphere combination during learning and implementation, separately.

The cross-stage consistency analysis with within-subject factorscondition*region*hemisphererevealed again an overall pattern similarity significant effect (Intercept, F[1,79] = 9.14, MSE = 0.00026, p = .003,${\hat{\eta }}_{p}^{2}=.104$). With a main effect of region (F[1,79] = 4.90, MSE = 0.00004, p = .030,${\hat{\eta }}_{p}^{2}=.058$), post-hoc estimated marginal mean comparison showed that S-R rules were represented consistently across stages more strongly in SPC versus IPC (t(79)=−2.21,p=.030) (Fig. S4bin the Supplementary Material). Additionally, region and hemisphere significantly interacted (F[1,79] = 6.39, MSE = 0.00002, p = .013,${\hat{\eta }}_{p}^{2}=.075$). Post-hoc the interaction revealed to be mainly guided by higher pattern similarity values in the right SPC as compared to the IPC (t(79)=−3.04,p=.003;Fig. 9b). There was no significant effect of condition (F[1.95,154.20] = 0.10, MSE = 0.00026, p = .900,${\hat{\eta }}_{p}^{2}=.001$) or hemisphere (F[1,79] = 1.33, MSE = 0.00004, p = .253,${\hat{\eta }}_{p}^{2}=.017$) on rule representation consistency across stages.

Overall, both prefrontal and parietal ROIs contained significantly decodable S-R rule representations, as suggested by the significant intercepts, without a significant difference between learning modes. Stronger pattern similarities were generally found in the learning versus implementation stage and more in the right than in the left hemisphere. Notably, the VLPFC contained consistent cross-stage representations, in contrast with the DLPFC.

##### 3.3.2.3 vPMC/IFJ and dPMC

**Fig. 10.**
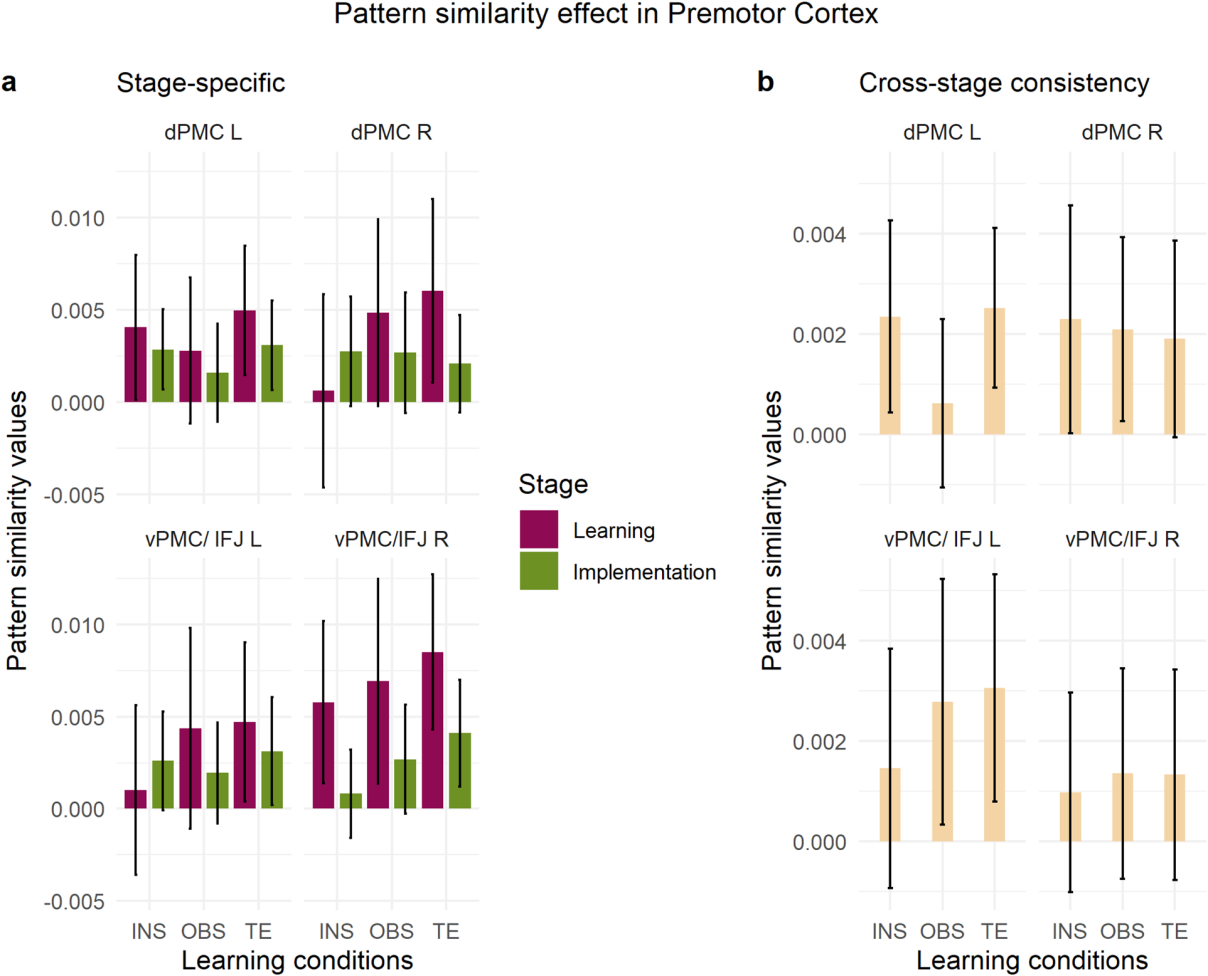
Pattern similarity effect in ventral and dorsal premotor cortex. 95% Confidence intervals are plotted. (a) Stage-specific pattern similarity analysis per each condition and stage. (b) Cross-stage consistency pattern similarity analysis per each condition. INS = instruction, OBS = observation, TE = trial-and-error. dPMC = Dorsal Premotor Cortex, vPMC/IFJ = Ventral Premotor Cortex/Inferior Frontal Junction.

The stage-specific pattern analysis with within-subject factorsstage*condition*region*hemisphererevealed a significant overall similarity pattern effect (Intercept, F[1,79] = 37.82, MSE = 0.00064, p < .001,${\hat{\eta }}_{p}^{2}=.324$). Particularly the right vPMC/IFJ appeared to contain significantly stronger pattern similarity values during learning as compared to implementation trials (3-way interactionstage*region*hemisphere, F[1,79] = 4.10, MSE = 0.00011, p = .046,${\hat{\eta }}_{p}^{2}=.049$;Figure 10a;Fig. S5ain the Supplementary Material). Importantly, individual S-R rule representations were significantly apparent across stages, regions, and hemispheres as suggested by testing the individual marginal means for the 3-way interactionstage*region*hemisphereagainst 0 (all individual interactions per eachstage*region*hemispherecombination, t[79]* > *2.39, p < .019). There was no significant main effect of condition (F[1.90,149.84] = 1.00, MSE = 0.00069, p = .368,${\hat{\eta }}_{p}^{2}=.012$), stage (F[1,79] = 2.86, MSE = 0.00069, p = .095,${\hat{\eta }}_{p}^{2}=.035$), region (F[1,79] = 0.89, MSE = 0.00025, p = .348,${\hat{\eta }}_{p}^{2}=.011$), or hemisphere (F[1,79] = 2.52, MSE = 0.00015, p = .116,${\hat{\eta }}_{p}^{2}=.031$).

A general pattern similarity effect was apparent from the cross-stage consistency analysis with within-subject factorscondition*region*hemisphere(Intercept, F[1,79] = 21.63, MSE = 0.00016, p < .001,${\hat{\eta }}_{p}^{2}=.215$), with no main effect of condition (F[1.96,154.53] = 0.11, MSE = 0.00021, p = .889,${\hat{\eta }}_{p}^{2}=.001$), region (F[1,79] = 0.08, MSE = 0.00006, p = .774,${\hat{\eta }}_{p}^{2}=.001$), or hemisphere (F[1,79] = 1.02, MSE = 0.00005, p = .315,${\hat{\eta }}_{p}^{2}=.013$), suggesting that stable cross-stage representations of individual S-R rules were on average similarly contained within all of the premotor ROIs across hemispheres and learning conditions (Fig. 10b;Fig. S5bin the Supplementary Material).

##### 3.3.2.4 Left motor ROI

**Fig. 11.**
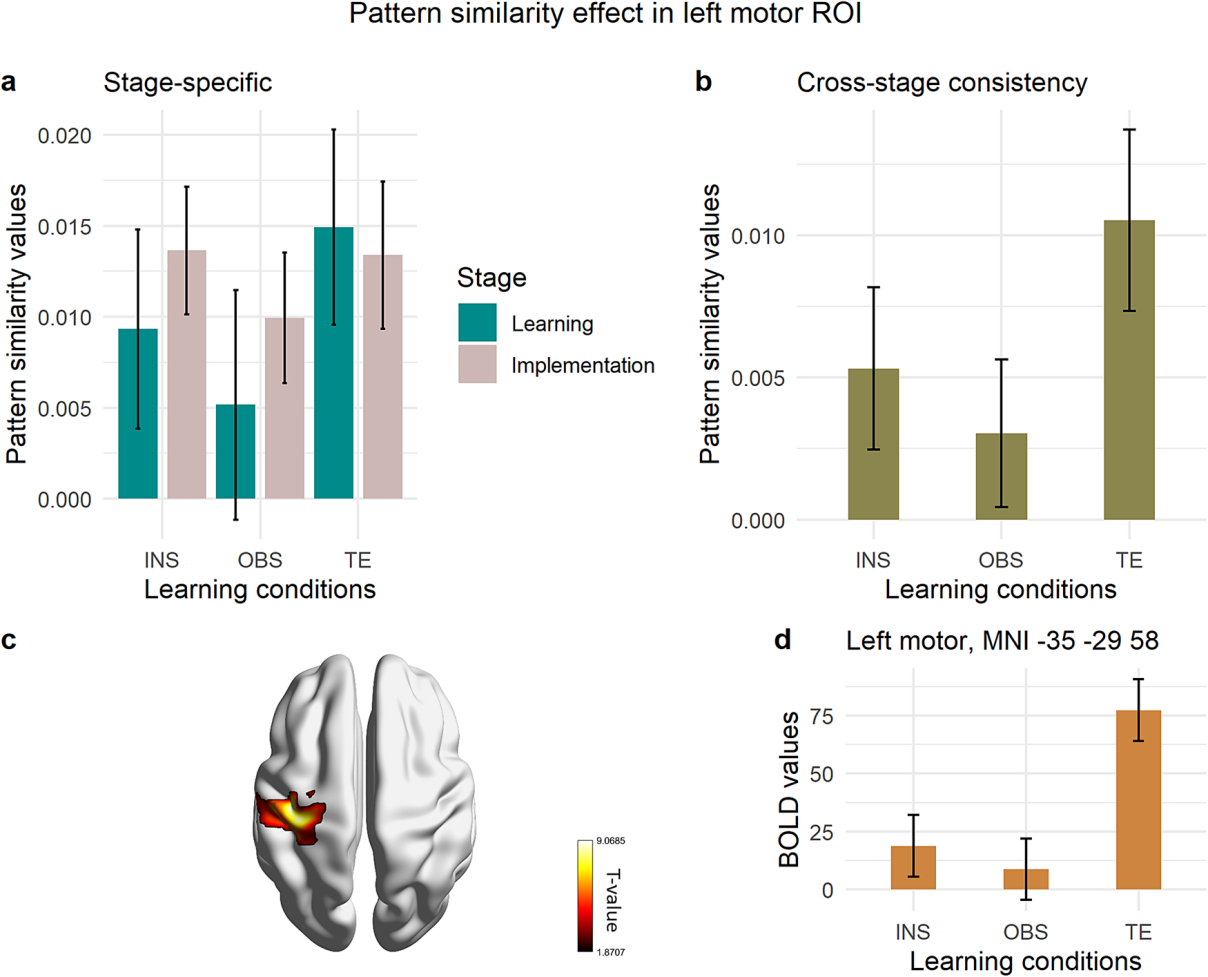
Pattern similarity effect in Left Motor ROI. 95% Confidence intervals are plotted. (a) Stage-specific pattern similarity analysis per each condition and stage. (b) Cross-stage consistency pattern similarity analysis per each condition. (c) Left motor cluster from conjunction analysis (FWE peak-level correction) for regions significantly active in trial-and-error more than instruction- and observation-based learning. (d) BOLD estimates plotted at the global maximum MNI coordinates [MNI -35 -29 58] of the left motor cluster as in (c). 90% confidence interval are plotted.

The group-level repeated-measures ANOVA with within-subject factors*condition*and*stage*revealed an overall significant pattern similarity effect in the left motor ROI (Intercept, F[1,79] = 105.92, MSE = 0.00055, p < .001,$\hat{\eta }=.573$). The ANOVA model additionally showed a significant effect of condition (F[1.89,149.02] = 4.69, MSE = 0.00040, p = .012,${\hat{\eta }}_{p}^{2}=.056$) with significantly stronger pattern similarities in trial-and-error as compared to observed trials (t(79)=−2.84,$p_{\text{MV}t(3)}\text{​}=.015$). The difference between the instruction and observation (t(79)=1.73,$p_{\text{MV}t(3)}\text{​}=.198$) and the instruction and trial-and-error conditions (t(79)=−1.41,$p_{\text{MV}t(3)}\text{​}=.337$) did not reach significance. Importantly, there was no interaction between stage and condition (F[1.97,155.72] = 1.00, MSE = 0.00050, p = .368,${\hat{\eta }}_{p}^{2}=.013$) as one could have expected from the response requirement in trial-and-error learning but not in instructed and observed learning trials, nor a significant effect of stage on motor representations pattern similarity (F[1,79] = 1.45, MSE = 0.00053, p = .233,${\hat{\eta }}_{p}^{2}=.018$). In fact, one could have predicted the implementation stage, which involved overt motor responses in all learning conditions, to yield significantly greater pattern similarity values compared to the learning stage, where learning was based on the passive view of S-R rules in the instruction- and observation-based learning conditions. Therefore, we inspected motor representations in the learning and implementation stages, for each condition separately. Pattern similarity values were significantly different from 0 across learning conditions in the implementation stage (trial-and-error,t(79)=6.59,$p_{\text{MV}t(6)}\text{​}<.001$; instruction,t(79)=7.74,$p_{\text{MV}t(6)}\text{​}<.001$; observation,t(79)=5.51,$p_{\text{MV}t(6)}\text{​}<.001$), as expected from the common motor response implementation across conditions. However, only trial-and-error and instructed but not observed trials led to pattern similarities values significantly different from 0 in the learning stage (trial-and-error,t(79)=5.53,$p_{\text{MV}t(6)}\text{​}<.001$; instruction,t(79)=3.39,$p_{\text{MV}t(6)}=.006$; observation,t(79)=1.63,$p_{\text{MV}t(6)}\text{​}=.476$).Figure 11ashows MVPA effect in the left motor ROI for each condition and stage. When testing for differences between learning conditions separately for each stage, there was a trend towards significance for trial-and-error > observation in learning trials (t(79)=−2.33,$p_{\text{MV}t(3)}\text{​}=.057$), which, we reasoned, could have been the factor driving the main effect of condition that we initially found (and that referred to pattern similarity values averaged over the levels of stage, that is, learning and implementation). Importantly, omitting correction for multiple comparisons led to a statistically significant difference between trial-and-error and observation in learning trials (t(79)=−2.33,p=.022). Meanwhile, the comparison between instruction and trial-and-error learning trials still yielded no significant difference (t(79)=−1.50,p=.138), confirming a comparable presence of decodable response information in motor cortex in instruction-based learning trials in the absence of overt motor response, as opposed to trial-and-error learning trials.

To sum up, the additional investigation of stage-wise pattern similarity effects within the left motor ROI showed that already during learning motor responses were represented in the absence of motor program enactment in instructed but not observed, as in our observation-based learning condition, trials. Additionally, motor responses were represented during learning in the same regions that contained pattern similarities during S-R rule implementation. This, we suggest, may reflect motor planning in the form of covert execution and motor imagery.

The cross-stage consistency analysis with within-subject factor*condition*showed an overall significant representational stability (F[1,79] = 46.79, MSE = 0.00020, p < .001,${\hat{\eta }}_{G}^{2}=.192$), with consistent cross-stage motor representations in instruction and trial-and-error, but not observation trials (t-test against 0 for instruction,t(79)=3.71,$p_{\text{MV}t(3)}\text{​}=.001$; trial-and-error,t(79)=6.56,$p_{\text{MV}t(3)}\text{​}<.001$; observation,t(79)=2.33,$p_{\text{MV}t(3)}=.065$). The repeated-measures ANOVA additionally revealed a main effect of condition (F[1.94,153.27] = 7.78, MSE = 0.00016, p ≤* .*001,${\hat{\eta }}_{G}^{2}=.056$), which post-hoc unfolded a significantly stronger cross-stage representational consistency in trial-and-error as compared to both instruction- (t(79)=−2.48,$p_{\text{MV}t(3)}\text{​}=.040$) and observation-based learning conditions (t(79)=−3.92,$p_{\text{MV}t(3)}\text{​}<.001$;Fig. 11b).

##### 3.3.2.5 Follow-up univariate analysis of motor activity

Despite the indisputable advantage of MVPA as compared to the standard GLM in detecting patterns of activity related to a certain cognitive process, we were interested in testing whether the rule representation-related left motor activation that was apparent from the MVPA was also detectable in the standard univariate analysis of learning trials. Our working hypothesis was that mere motor preparation in the absence of overt motor implementation, as apparent from the multivariate pattern similarity analysis in instruction-based learning during the learning stage, would be associated with drastically weaker activity in the univariate analysis as compared to trial-and-error learning trials, which required motor implementation. This hypothesis was additionally guided by Ariani et al.’s results (2022) in showing that motor preparation was associated with effector-specific MVPA but not univariate effects in the motor cortex. Therefore, we were interested in finding a left motor activity cluster that we could make sure was related to response preparation, like in the trial-and-error learning condition, and in testing whether this was significantly activated during instructed and observed learning trials, which did not require response implementation during learning.

We implemented (mean) across-repetition t-contrasts for differences between conditions and a conjunction analysis testing for brain regions with increased BOLD signal in trial-and-error versus instruction and trial-and-error versus observation (cf. global null for congruent contrasts,Friston et al., 2005) during the learning stage. Reported tables and figures show results after FWE peak level correction. We corrected at the peak and not cluster level for cluster-specific visualization purposes. The analysis showed a significant left motor cluster with global maximum at MNI -35 -29 58 (Fig. 11c;Table 9). BOLD estimate values at the global maximum peak coordinates, showed, in line with our hypothesis, a very weak motor activation during learning when response implementation was not required as compared to trial-and-error learning (Fig. 11d).

**Table 9.** Table of the left motor cluster activation from the conjunction analysis testing for regions significantly active in trial-and-error >instruction- and observation-based learning.

	MNI coordinates				
Region	x	y	z	Extent (voxels)	t-value (peak)
Precentral_L	-35	-29	58	5061	9.07
	-25	-19	78		6.00
Rolandic_Oper_L	-49	-23	20		8.29
Postcentral_L	-63	-15	32		4.38
Parietal_Inf_L	-57	-23	50		8.62
Putamen_L	-23	6	-5		4.60
Insula_L	-39	-3	14		7.55

T = 3.27, peak-level FWE-correction (p < .05). Regions labeled using the AAL3 atlas. Peak separation threshold = 20 mm. Regions are organized anatomically by cluster, starting from the maximal t-value.

Similar results were observed at distinct MNI coordinates within the left motor cluster, both from the whole-brain searchlight analysis on implementation trials and the brain map depicting heightened activity during S-R rule motor execution across repetition levels and learning conditions.

Together, these results suggest that covert motor preparation in the absence of overt motor implementation during learning, as in our instruction- and observation-based learning conditions, is associated with drastically weaker left motor activity in the univariate analysis as compared to trial-and-error trials, which required motor implementation during learning. This contrasts with the multivariate pattern analysis, which showed statistically similar rule decodability in instruction-based and trial-and-error learning during the learning stage. Our results are in line with the recent work byAriani et al. (2022), who showed the advantage of the multivariate approach in unfolding covert motor preparation in the absence of motor implementation. In turn, this seems to provide firm evidence for motor preparation, that is, proceduralization, already during the mere instruction phase.

##### 3.3.2.6 Motor and kinesthetic representations of instructed response in somatosensory cortex

Importantly, one of the reviewers pointed us to a recent paper byPalenciano et al. (2023)where regions specific to action and its somatosensory consequences (i.e., action effects), as detected by specific localizer tasks, activated overlapping brain patterns of activation, which were, in turn, preactivated during the instruction preparation period. As suggested byPalenciano et al. (2023), the difference between trial-and-error and instruction-based learning trials in the follow-up univariate analysis on motor activity could plausibly be driven not only by motor implementation during learning but also by action effects.

**Fig. 12.**
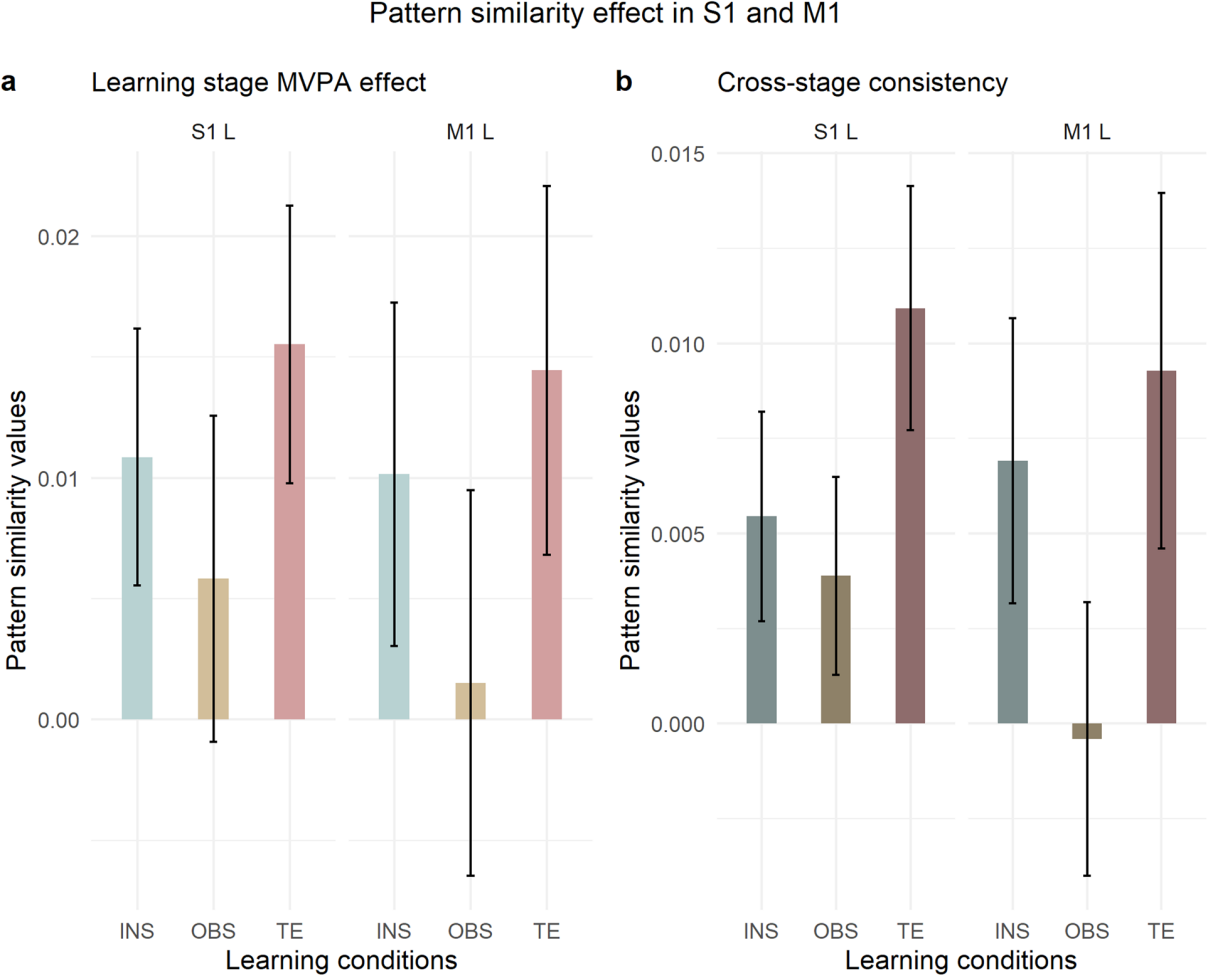
Pattern similarity effect in S1 and M1. 95% Confidence intervals are plotted. (a) Stage-specific pattern similarity analysis per each condition and stage. (b) Cross-stage consistency pattern similarity analysis per each condition. INS = instruction, OBS = observation, TE = trial-and-error. S1 = primary sensory cortex; M1 = primary motor cortex. S1 was defined as BA 1, 2, and 3 intersected with the searchlight brain map across conditions in implementation trials. M1 was defined as BA 4 intersected with the searchlight brain map across conditions in implementation trials. BA = Brodmann area.

To explore this further, specifically when response execution is not required for learning as in our instruction condition, we implemented an additional multivariate analysis within the left primary motor cortex, M1, and primary sensory cortex S1 (Fig. 12). Via the SPM12 toolbox WFU PickAtlas (Maldjian et al., 2003,^2004^), we extracted Brodmann areas 1, 2, and 3 for S1, and Brodmann area 4 for M1 in the left hemisphere (Lancaster et al., 1997,^2000^) and we intersected each ROI, separately, with the motor ROI extracted from the searchlight across conditions on implementation trials (SPM12 software package, Statistical Parametric Mapping, RRID:SCR_007037; Matlab, version R2020b;The MathWorks Inc., 2020; RRID:SCR_001622). For the stage-specific analysis, we only included learning trials, with the hypothesis that comparable activation patterns should be observed in both S1 and M1 in the instruction and trial-and-error conditions, possibly reinforcing our previous results in the searchlight-related ROI MVPA. Particularly for instructed learning trials, an MVPA effect in motor and sensory regions would possibly reflect motor and kinesthetic representations of the instructed response triggered by covert response simulation during learning. Additionally, we implemented a cross-stage analysis to test the consistency between the learning-related representations and those elicited during implementation trials.

The stage-specific pattern similarity analysis on learning trials, which included the within-subject factors*condition*, and*region*, revealed a significant overall pattern similarity effect (Intercept, F[1,79] = 27.03, MSE = 0.00168, p < .001,${\hat{\eta }}_{p}^{2}=.255$). There was no main effect of region (F[1,79] = 1.43, MSE = 0.00034, p = .235,${\hat{\eta }}_{p}^{2}=.018$) on pattern similarity values, suggesting that comparable representations were instantiated in both M1 and S1. However, the model revealed a significant main effect of condition (F[1.94,153.38] = 3.56, MSE = 0.00150, p = .032,${\hat{\eta }}_{p}^{2}=.043$), with post-hoc tests showing significantly higher pattern similarity in the trial-and-error condition compared to observation-based learning (t(79)=−2.47,$p_{\text{MV}t(3)}\text{​}=.041$). There were no statistical differences between instruction- and observation-based learning (t(79)=1.61,$p_{\text{MV}t(3)}\text{​}=.247$) or instruction and trial-and-error learning (t(79)=−1.14,$p_{\text{MV}t(3)}\text{​}=.494$). We additionally computed one-sample t-tests to check whether pattern similarity values during learning were significantly greater than 0 for each condition within S1 and M1, separately. During learning trials, the MVPA effect was found to be non-significantly different from 0 only in the observation condition in both S1 (OBS,t(79)=1.72,$p_{\text{MV}t(2)}\text{​}=.143$; INS,t(79)=4.07,$p_{\text{MV}t(2)}\text{​}<.001$; TE,t(79)=5.37,$p_{\text{MV}t(2)}\text{​}<.001$) and M1 (OBS,t(79)=0.38,$p_{\text{MV}t(2)}=.881$; INS,t(79)=2.84,$p_{\text{MV}t(2)}\text{​}=.011$; TE,t(79)=3.77,$p_{\text{MV}t(2)}\text{​}<.001$).

The cross-stage consistency analysis with factors*region*and*condition*revealed an overall significant MVPA effect (Intercept, F[1,79] = 35.85, MSE = 0.00048, p =*< .*001,${\hat{\eta }}_{p}^{2}=.312$). A main effect of condition was observed (F[1.86,146.67] = 7.78, MSE = 0.00039, p =*< .*001,${\hat{\eta }}_{p}^{2}=.090$), with a significant difference between trial-and-error and observation-based learning (t(79)=−4.20,$p_{\text{MV}t(3)}\text{​}<.001$). No significant differences were found between the other conditions (INS-OBS,t(79)=2.28,$p_{\text{MV}t(3)}\text{​}=.063$; INS-TE,t(79)=−1.64,$p_{\text{MV}t(3)}\text{​}=.234$). Notably, without correction for the number of tests, the difference in cross-stage representation consistency between instructed and observed trials was significant (INS-OBS,t(79)=2.28,p=.025). There was no main effect of region (F[1,79] = 2.14, MSE = 0.00012, p = .147,${\hat{\eta }}_{p}^{2}=.026$); however, there was a significant interaction between condition and region (F[1.95,153.98] = 4.22, MSE = 0.00008, p = .017,${\hat{\eta }}_{p}^{2}=.051$). Post-hoc tests revealed that within S1, trial-and-error learning showed a significantly greater cross-stage consistency compared to both instruction- (TE-INS,t(79)=−2.63,$p_{\text{MV}t(3)}\text{​}=.027$) and observation-based learning (TE-OBS,t(79)=−3.58,$p_{\text{MVt}(3)}\text{​}=.002$). No significant difference was observed in S1 between instruction- and observation-learning related representations (t(79)=0.87,$p_{\text{MV}t(3)}\text{​}=.657$). In M1, on the other side, both trial-and-error and instruction conditions showed significantly greater consistency compared to observation (TE-OBS,t(79)=−4.05,$p_{\text{MV}t(3)}\text{​}<.001$; INS-OBS,t(79)=2.89,$p_{\text{MV}t(3)}\text{​}=.013$), whereas there was no significant difference between trial-and-error and instruction-based learning on representational consistency (t(79)=−0.77,$p_{\text{MV}t(3)}\text{​}=.716$).

## 4 General Discussion

The present study investigated the neural and representational dynamics at play during S-R associative learning in different learning modes. We were first of all interested in extending previous research on instructed rapid learning to the investigation of the instruction period as opposed to the first implementation trials after instruction. Furthermore, we took into examination how this compares to learning by trial-and-error. Via the inclusion of a “mid-way condition” that shared features with both instruction-based and trial-and-error learning, we were able to dissociate motor preparation from motor implementation and better understand the role that these play during learning.

In the following section, we initially explore the advantage of instruction-related behavior in learning compared to the other learning modes, focusing on the individual motor response representations observed during the instruction period in the absence of overt motor response implementation. Subsequently, we delve into the fMRI univariate analysis findings, which revealed a common frontoparietal decrease and DMN increase across conditions during learning, along with signal changes specific to instruction-based and trial-and-error learning trials. This is examined in the context of cognitive control demand. Lastly, we examine the findings from the ROI-based MVPA in prefrontal, parietal, and premotor ROIs, particularly discussing the consistency of brain representations across most ROIs in terms of early representations guiding later task performance.

### 4.1 The advantage of explicit instructions on learning: early proceduralization of instructed S-R rules

Behaviorally, we confirmed previous findings on the advantage during later task execution that explicit instructions offer when learning novel S-R rules as compared to rules that are explored via trial-and-error (and, in our design, via observation of correct and incorrect mappings). This, as previously assumed, points towards a detrimental effect of error processing, prominent in our trial-and-error and observation-based learning conditions, on learning successive correct S-R rules (Li et al., 2011;Monsell & Graham, 2021; cf.Ruge, Karcz, et al., 2018). Furthermore, it suggests that overt response implementation in trial-and-error learning, as opposed to merely observing correct and incorrect rules and feedback, mitigates the negative effect of error processing during learning on the subsequent implementation trials. To elaborate, both response accuracy as well as response speed in implementation trials in the trial-and-error condition showed values that fell between those observed in the instruction-based learning condition, where performance was at its peak, and the observation-based learning condition, where performance was at its lowest (Fig. 3b). In our experimental design, the key distinction between learning via trial-and-error and mere observation was the requirement for overt response implementation in the former but not the latter. Therefore, we reasoned that the overall better performance in trial-and-error implementation trials, in contrast to previously observed implementation trials, may be attributed to learning via overt repeated attempts rather than the passive observation of correct and incorrect S-R links and feedback. The mechanism underlying this advantage of overtly implementing versus merely observing correct and incorrect links might involve a representational update in declarative as well as procedural WM at each incorrect feedback following an incorrectly implemented S-R link. This representational update could serve as a reset mechanism, potentially aiding the learning process. This hypothesis is supported by the significantly greater pattern similarity effect in trial-and-error compared to observed trials, as observed in the stage-specific pattern analysis of the left searchlight-derived motor ROI (Fig. 11a), as well as in the additional multivariate analysis within M1 and S1 (Fig. 12a). In both analyses, upon closer examination, we found that the pattern similarity effect in observed learning trials was not statistically significant as opposed to the other two learning conditions. This suggests that representations of individual motor programs and their effects (Palenciano et al., 2023) are not represented in the left motor and somatosensory cortices during passive observation of correct and incorrect rules and feedback. Instead, our results suggest that for motor and somatosensory representations to be activated within the motor and sensory cortices when processing incorrect trials for learning, overt response implementation is a necessary condition. This contrasts with the instruction-based learning condition, where only correct S-R links were provided during learning. The stage-specific pattern analysis within the searchlight-derived motor ROI, as well as within M1 and S1, in fact, revealed a significant pattern similarity effect already during instructed learning trials and in the absence of overt response implementation. This suggests that covert motor preparatory mechanisms are at play early during instructed learning trials and before response implementation, which, in turn, might explain the behavioral advantage of instruction-based learning over the other learning modes, especially in light of the significantly faster RTs in implementation trials (Fig. 3b). Notably, motor and somatosensory representations observed during instructed learning trials were found in the somatomotor regions that contained motor and somatosensory representations during implementation trials. This suggests that preparatory mechanisms during instruction-based learning occur in the same left somatomotor regions where motor programs, but also their somatosensory effects, are represented during implementation trials (cf.Ariani et al., 2022;Palenciano et al., 2023). If during implementation trials the pattern similarity effect in left somatomotor regions is likely to reflect overt motor implementation, on the other hand, during instructed learning trials it is plausible to assume that this mirrored covert motor implementation and motor imagery. The presence of somatomotor representations during instructed learning trials in the same somatomotor regions that contained information during implementation trials is further complemented by the overlap that these representations showed in the cross-stage consistency analysis in the searchlight-derived motor ROI, as well within M1 and S1. It should be noted that the additional analysis within M1 and S1, particularly the cross-stage analysis, revealed a dissociation in observation-based learning between S1 and M1 (cf.Fig. 12). One possible explanation for this inconsistency could be, as previously discussed in the context of the LPFC, a higher sensibility of the cross-stage analysis (cf.Section 3.3.2). However, for the observation-based learning condition further research is necessary to draw specific conclusions and these are outside of the scope of the present paper. We can only conclude that in our observation-based learning condition, during learning trials, weak preparation processes took place in guiding later performance. Additionally, it is important to highlight that a significantly stronger representational consistency between the learning and the implementation stage in our searchlight-derived motor ROI MVPA, as well as in S1, was observed when overt motor implementation was required for learning, as in the trial-and-error learning condition. This suggests that a stronger representational consistency between the learning and the implementation stage requires overt response execution, and that covert versus overt response implementation leads to motor representations that are less consistent with those observed in the implementation stage.

In summary, these findings together suggest that under explicit instructions, procedural representations are formed as early as during learning and before implementation in the same somatomotor regions that will later support response implementation, possibly guiding later performance during the implementation stage.

Last, based on recent findings (Palenciano et al., 2023) that action effects are represented not only in S1, but also in M1, one of the reviewers pointed out that the difference between instructed and trial-and-error learning trials within M1 could be driven not only by actual motor execution, but also by its somatosensory effects in the trial-and-error condition. In the stage-specific analysis within the searchlight-related motor ROI, we did not observe a statistical difference between the trial-and-error and instruction-based learning conditions, although the MVPA effect in instructed learning trials was still numerically lower than in trial-and-error learning trials (cf.Fig. 11a). This contrasts with our follow-up univariate analysis of motor activity that showed a drastically weak BOLD signal in instructed as compared to trial-and-error learning trials (cf.Fig. 11c). In this latter context, the lack of response execution, as well as its somatosensory consequences, could have driven the difference between trial-and-error and instructed learning trials motor-related BOLD signal. This additional analysis complementsPalenciano et al. (2023)andAriani et al. (2022)’s findings with respect to the power of MVPA in detecting patterns of activity related to covert response preparation.

### 4.2 Mean neural change during learning reflects decreasing cognitive control demand

The univariate analysis confirmed previous findings showing that key regions of the DMN increased in activity early during learning and across all learning modes. The DMN increase during learning was accompanied by a frontoparietal decrease along the inferior parietal sulcus and frontal midline, possibly mirroring lower cognitive control demand as learning proceeded. During implementation trials, the decrease was constrained to motor regions including postcentral gyrus/inferior parietal, vermis and cerebellum VI, left thalamus, and putamen, possibly supporting mechanisms related to visuo- and somatomotor adaptation (e.g.,Floyer-Lea & Matthews, 2004,Makino et al., 2016for a review).

#### 4.2.1 The role of the caudate head/ventral striatum in instruction-based learning trials

When inspecting linear signal change per each stimulus repetition that was specific to one learning mode as compared to the others, we found activity patterns specific to instruction and trial-and-error, but not to observed trials. One reason could logically be that our observation-based learning condition shared features with both instruction-based- and trial-and-error learning. Thus, it may be plausible to assume that observation-based learning in our paradigm was supported by neural mechanisms common to both instruction-based and trial-and-error learning.

Consistent withRuge et al. (2019), where the ventral striatum was found to increase from the first implementation trial after instruction, we observed a similar signal increase in the instruction-based learning condition. However, in the present study, we further showed that this increase occurs early during learning, in the absence of overt response implementation. Additionally, we confirmed the specificity of this signal change to learning via explicit instructions in contrast with trial-and-error and observation-based learning.

The ventral striatum has been linked to reward processing, including positive feedback (e.g.,Schott et al., 2007; for an overview,Daniel & Pollmann, 2014), and novel rule formation (Bédard & Sanes, 2009). In line with this, we observed relatively high activity in this region during trial-and-error learning trials, even at the initial repetition levels during learning. We hypothesized that this heightened activity in the ventral striatum during trial-and-error learning may be a result of immediate responsiveness to feedback-driven reward processing, especially when the correct response is overtly executed, as is the case in trial-and-error learning. This is in contrast to observed and instructed learning trials, where no overt execution of responses was required during the learning phase and, consequently, where we observed lower initial ventral striatal activity. During instructed learning trials, although no feedback is provided, we reasoned that covert response implementation, as indicated by the pattern similarity effect in the left motor ROI, likely occurs more frequently as the learner encounters the stimulus repeatedly. Thus, this gradual increase in covert response implementation might drive the incremental rise in ventral striatal activity from one repetition to the next that we observed in instructed trials (cf.Pasupathy & Miller, 2005).

Other regions that exhibited greater increases during instruction than during observation and trial-and-error learning included extended clusters in the right hemisphere, encompassing important hubs of the DMN such as the angular gyrus, precuneus, and ventromedial prefrontal cortex (VMPFC). We postulate that the overall simplicity of attending explicit instructions, coupled with the absence of ongoing interference caused by error trials in comparison to the other learning conditions, potentially led to a more rapid decrease in cognitive control demand. This reduction in demand likely manifested as a greater engagement of DMN-hub regions, which in our paradigm was more apparent in the right hemisphere under the instruction condition. This contrasts with the common bilateral DMN engagement observed across learning modes. Consistent with this rationale, when examining regions that exhibited a greater decrease in activity during trial-and-error learning compared to instructed and observed learning trials, results implicated regions associated with cognitive control demand, such as the DLPFC and anterior insula (e.g.,Molnar-Szakacs & Uddin, 2022;Wu et al., 2019). Notably, these regions displayed relatively lower activity levels during instructed trials (Fig. 6c), supporting the hypothesis that learning through explicit instructions places less cognitive control demand.

#### 4.2.2 Cingulo-opercular network and active rule exploration in trial-and-error learning

During trial-and-error learning trials, as opposed to the other conditions, we observed a greater activity decrease in the bilateral anterior insula (AI) and the bilateral anterior portion of the mid-cingulate cortex (aMCC, in the literature often referred to as dorsal or supracallosal anterior cingulate cortex;Fig. 6b). These regions commonly co-activate as key components of the cingulo-opercular network (CON), known for its role in goal-directed cognitive control (Dosenbach et al., 2007,^2008^;Molnar-Szakacs & Uddin, 2022;Wood & Nee, 2023;Wu et al., 2019). The AI and the aMCC are suggested to support intentional action (Brass & Haggard, 2010;Hoffstaedter et al., 2014), evaluating action-outcomes to reinforce behavioral responses associated with more desirable results. One hypothesis is that the AI, with its rich connectivity to sensory areas (Dionisio et al., 2019;Gogolla, 2017), generates a “salience map” (Michel, 2017) of behaviorally relevant events (Brass & Haggard, 2010;Han et al., 2019;Hoffstaedter et al., 2014;Menon & Uddin, 2010;Touroutoglou et al., 2020) towards which attentional and WM resources (Hausman et al., 2021) can be directed for further processing and response (via the mACC,Han et al., 2019;Molnar-Szakacs & Uddin, 2022;Touroutoglou et al., 2020). This assessment influences the ability to transition between tasks and rules (Dosenbach et al., 2007,^2008^;Hausman et al., 2021;Menon & Uddin, 2010). In trial-and-error learning, this translates into evaluating stimulus-response-feedback and shifting between different responses for the same stimulus until the correct S-R mapping is implemented. This attentional shift ultimately contributes to forming and adjusting behavioral strategies, crucial for successful trial-and-error learning (Mohr et al., 2018). We propose that in trial-and-error learning, the CON supports the disengagement from previous error trials to facilitate the shift towards new S-R mappings, as well as the formation and implementation of adaptive and efficient learning strategies. Importantly, according to our hypothesis, conditions that do not require to shift attentional resources from one mapping to another for the same stimulus, or necessitate response implementation and subsequent behavioral adjustment, should not elicit a comparable signal pattern in the CON-associated areas. In line with this, the instruction-based learning condition showed low and stable activity in both AI and aMCC (Fig. 6c). The observation-based learning condition, on the other hand, plausibly required a representational shift akin to trial-and-error. However, the passive observation, as opposed to the active exploration of correct and incorrect rules during trial-and-error learning, plausibly mitigated the CON engagement, leading to lower initial activity in these regions (Fig. 6c). Consistent with this reasoning, conditions that did not require motor implementation showed low activity in the aMCC, which supports top-down intentional movement with its connection to premotor, prefrontal, and parietal areas, as well as the cerebellum (Dosenbach et al., 2007;Hoffstaedter et al., 2014). Notably, these regions also decreased in activity during trial-and-error learning, possibly indicating that the activity in these areas decreased as a result of task automatization as learning progressed.

We considered two additional trial-and-error features that possibly drove a greater initial CON engagement as well as a subsequent signal decrease in trial-and-error learning. CON-related regions are sensitive to the level of uncertainty (or entropy,Fan, 2014;Wu et al., 2019), which plausibly was maximal at the beginning of trial-and-error learning, before any motor response was implemented, and gradually decreased via active rule exploration and the convergence towards predictable and accurate S-R mappings (Mohr et al., 2018). Importantly, this contrasts with the observation-based learning condition, which plausibly did not trigger the same level of uncertainty as trial-and-error learning because participants were not actively engaged in the motor response or the decision-making process. In other words, trial-and-error learning was the only condition in our study requiring context-dependent behavioral adjustment, which is supported by the CON as previously explained. Finally, AI and aMCC are sensitive to the rate of information under time constraints (Wu et al., 2019). We reasoned that the rate of information processing in trial-and-error learning plausibly was likely heightened as participants needed to rapidly cycle through the different previously incorrect implemented responses to identify (and implement within a certain time frame) the correct mapping. One hypothesis is, therefore, that the additional working-memory resources required for retrieving previous incorrect trials, coupled with the motor response requirement in trial-and-error learning, elicited a heightened CON activity and subsequent decrease in this condition.

### 4.3 Representational dynamics in prefrontal, parietal and premotor cortex

Our approach to the multivariate analysis was twofold. First, we aimed to identify brain regions where a pattern similarity effect could be detected across learning modes and stages. Once we established that individual rules were indeed represented in the brain, we employed an ROI-based approach. This enabled us to delve into more fine-grained questions about differences between regions, conditions, and stages. Importantly, the present study built uponRuge et al. (2019)in extending the investigation of first implementation trials after instruction to the exploration of the initial learning phase. A key objective was to track the representational dynamics during S-R learning and implementation and assess the similarity between these representations as they transitioned from the learning to the implementation stage. Notably, this particular analysis was not feasible in the design inRuge et al. (2019). To elaborate, finding rule information within the same brain region during both the learning and implementation stages, when pattern similarities are calculated separately within each stage, does not imply that these representations are similar. To address this, we employed a cross-stage consistency analysis, which allowed us to test for overlapping representations between learning and implementation trials in two distinct scenarios. The first scenario, exemplified by the trial-and-error condition, pertains to cases where response implementation was required early in the learning process. In contrast, the second scenario applies to learning conditions that did not require response implementation during the learning phase. In these instances, the transition to implementation trials was more profound and can be viewed as the shift from non-executed to executed trials. This latter scenario was particularly relevant to our instruction- and observation-based learning condition.

Rule information was identified in all predefined ROIs, encompassing the bilateral prefrontal and parietal cortices, as well as the bilateral premotor and left motor cortices. The presence of S-R rule information in frontoparietal regions is consistent with the role of these areas in exerting sustained attention and actively holding relevant information in WM (Chai et al., 2018;Cole et al., 2013;Dosenbach et al., 2007,^2008^;Ekman et al., 2016;Marvel et al., 2019). We observed stronger pattern similarity effects during learning as compared to implementation trials in prefrontal and parietal ROIs. This fits well with the typical frontoparietal mean activity decrease as learning progresses that we observed in the present study as well as in previous investigations (Bédard & Sanes, 2009;Hampshire et al., 2016,^2019^;Mohr et al., 2016;Ruge & Wolfensteller, 2010,^2013^).

During implementation trials, the pattern similarity effect in both the VLPFC and DLPFC did not reach statistical significance. This contrasts withRuge et al. (2019), in which individual rules were found to be represented specifically in the left VLPFC during early implementation trials following instruction. However, our design contrasts with that ofRuge et al. (2019)in the way we tracked the whole learning stage. In fact, inRuge et al. (2019), instructions were presented once and they were directly followed by implementation trials. Hence, it is plausible to assume that participants were still consolidating the one-time instructed rules at the first implementation trials after first-time instruction. This contrasts with our experimental design where each instructed S-R link was presented 4 times during the learning stage. Therefore, by the start of the implementation stage, rules were already learned, as apparent from the high behavioral accuracy in implementation trials. This might explain the low pattern similarity effect in prefrontal regions during implementation trials. In the parietal cortex, rules were more strongly represented in the superior part, which has been previously linked to information manipulation in WM (Crone et al., 2006;Koch et al., 2005;Koenigs et al., 2009;Wager & Smith, 2003). Notably, both the superior and inferior parts of the parietal cortex, as well as the VLPFC, exhibited overlapping rule representations across both the learning and implementation stages. This observation implies that these regions play a crucial role in constructing initial task representations (cf.Cole et al., 2011;González-García et al., 2017;Muhle-Karbe et al., 2017;Woolgar et al., 2016;Yang et al., 2019) during early learning trials, whether they involve overt execution as in trial-and-error or not as in instruction- and observation-based learning. Moreover, they seem to maintain this information consistently during rule execution, potentially aiding performance in subsequent rule implementation. This hypothesis was already proposed byRuge et al. (2019). However, as explained before, representational consistency testing was not possible in the previous design. We, therefore, confirmed the early formation of abstract rule representation in VLPFC and DLPFC, as well as in the parietal cortex, and the maintenance of these very same representations throughout S-R implementation.

In the premotor ROIs, a significantly greater pattern similarity effect in learning versus implementation trials was uniquely observed in the right vPMC/IFJ. This region is associated with abstract rule representation and rule updating (Brass & von Cramon, 2002,^2004^;Derrfuss et al., 2004,^2005^;Muhle-Karbe et al., 2017). Accordingly, the right IFJ has been found to contain decodable rule information during the delay period following instructions that had to be implemented later on as opposed to instructions that had to be simply memorized (Muhle-Karbe et al., 2017). This, together with the IFJ’s selective response to instruction cues before task implementation (Brass & von Cramon, 2002, 2004), suggests a role of this region in task preparation and might explain the greater similarity effect during learning versus implementation trials. Individual rules were similarly represented across stages in the dPMC, a region associated with motor imagery (Pilgramm et al., 2016;Zabicki et al., 2019), as well as movement planning and execution (Hoshi & Tanji, 2007). Rule information in these regions likely mirrors preparatory mechanisms in the presence and absence of motor implementation during learning and overt motor execution during rule implementation. The premotor individual rule representations were similar from the learning to the implementation stages, suggesting that the premotor cortex is not only involved in preparatory mechanisms early during learning but maintains rule information during execution, possibly supporting rule implementation. Notably, the premotor cortex has been associated with information rehearsal in WM and it has been proposed to support the WM system beyond the traditional prefrontal and parietal regions (Marvel et al., 2019). This is consistent with the overlap of individual rule representations not only in frontoparietal but also in premotor cortices, supporting the idea that all these regions collectively contribute to maintaining rule information actively from learning to rule execution, possibly to guide task performance.

## 5 Conclusions

In the present study, we built upon prior investigations that focused on the initial implementation trials following first-time instruction. With a focus on the initial learning of S-R links via instruction and trial-and-error, we replicated and extended established results regarding the reorganization of brain activity in frontoparietal and DMN-related regions during learning (and not only implementation) trials. Crucially, the novelty of our study particularly lies in the exploration of individual rule identity representations during the critical transition from learning to implementation. Our study’s innovation lies in uncovering the early emergence of individual rule representations in prefrontal, parietal, premotor, and left motor cortices. Notably, these early representations align with those observed during subsequent implementation trials. This compelling observation suggests that abstract and procedural representations take shape during the early stages of learning, even in the absence of overt motor execution, and persist throughout motor implementation, possibly playing a crucial role in guiding successful task performance.

## Supplementary Material

Supplementary Material

## Data Availability

Group-level images, as well as ROI-based MVPA and behavioral data are available athttps://osf.io/5mcf8/.

## Footnotes

* Copyright Waiver. The above boilerplate text was automatically generated by fMRIPrep with the express intention that users should copy and paste this text into their manuscripts*unchanged*. It is released under the CC0 license.
