# Correction to: Initial learning in the brain: From rules to action

**DOI:** 10.1162/imag_x_00375

**Published:** 2024-11-08

**Authors:** Sofia Fregni, Uta Wolfensteller, Hannes Ruge

**Affiliations:** Fakultät Psychologie, Technische Universität Dresden, Dresden, Germany

In the original publication, there was an error in the reporting of confidence intervals (CIs) in three subplots: Figure 4c, Figure 6c, and Figure 11d. Specifically, the CIs presented in these plots were not divided by two, resulting in an overestimation of the intervals displayed.The corrected figures reflect this adjustment, with each CI inFigures 4c,6c, and11dnow accurately halved. This correction does not affect the overall interpretation of the results but ensures that the reported CIs accurately represent the intended statistical estimates.

**Fig. 4.**
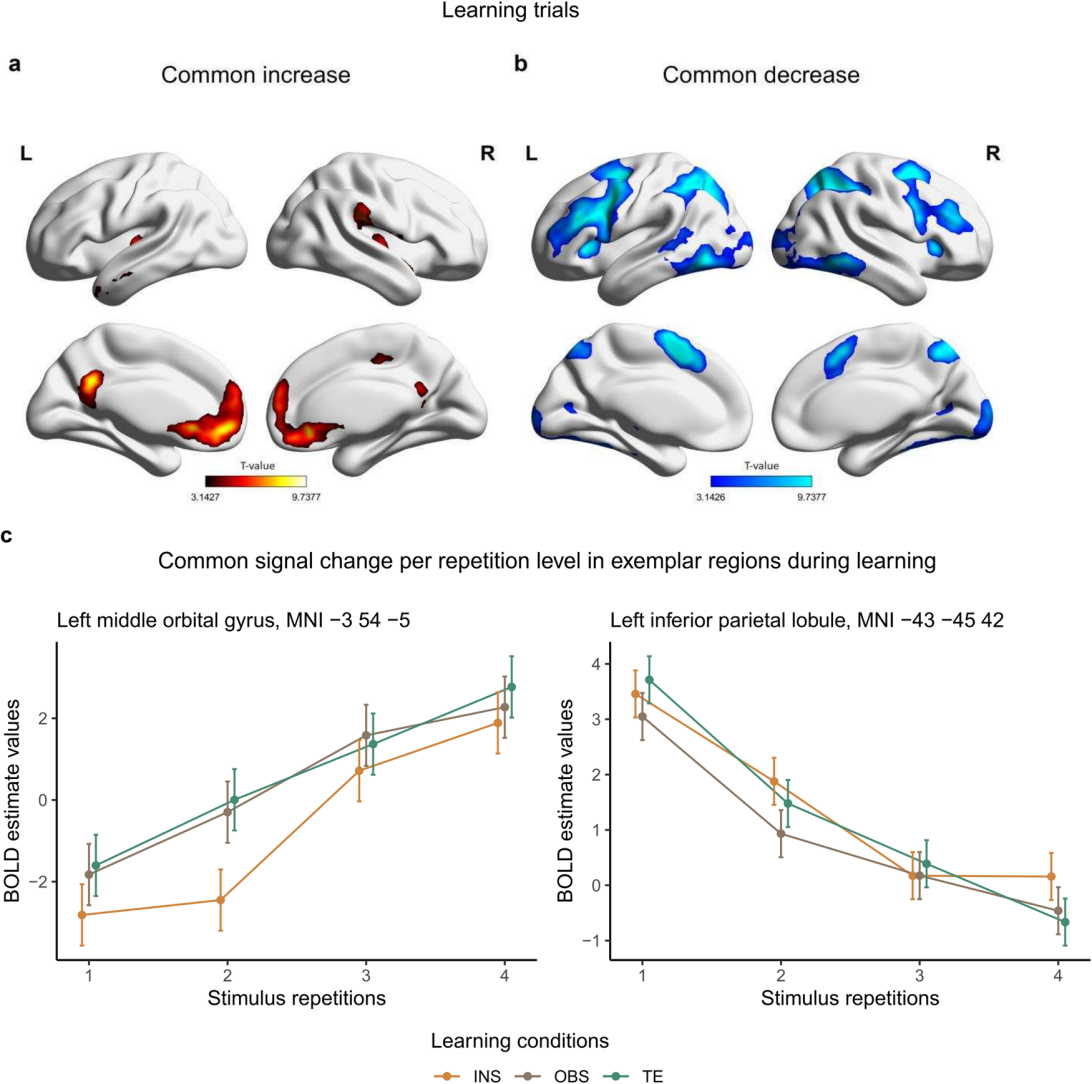
Across-condition signal change during S-R learning. (a) Medial and lateral views of T-maps exhibiting increasing activity across learning conditions. (b) Medial and lateral view of T-maps exhibiting decreasing activity across learning conditions. (c) Stimulus-repetition-wise signal increase and decrease per each learning conditions (INS = instruction, OBS = observation, TE = trial-and-error) and stimulus repetition in exemplar brain regions. 90% confidence interval are plotted. T-maps and BOLD estimate values result from the conjunction analysis (conjunction null) testing for brain regions with stimulus-repetition-wise signal increase or decrease common to all conditions during S-R learning. Results are FWE-cluster corrected (p < .05).

**Fig. 6.**
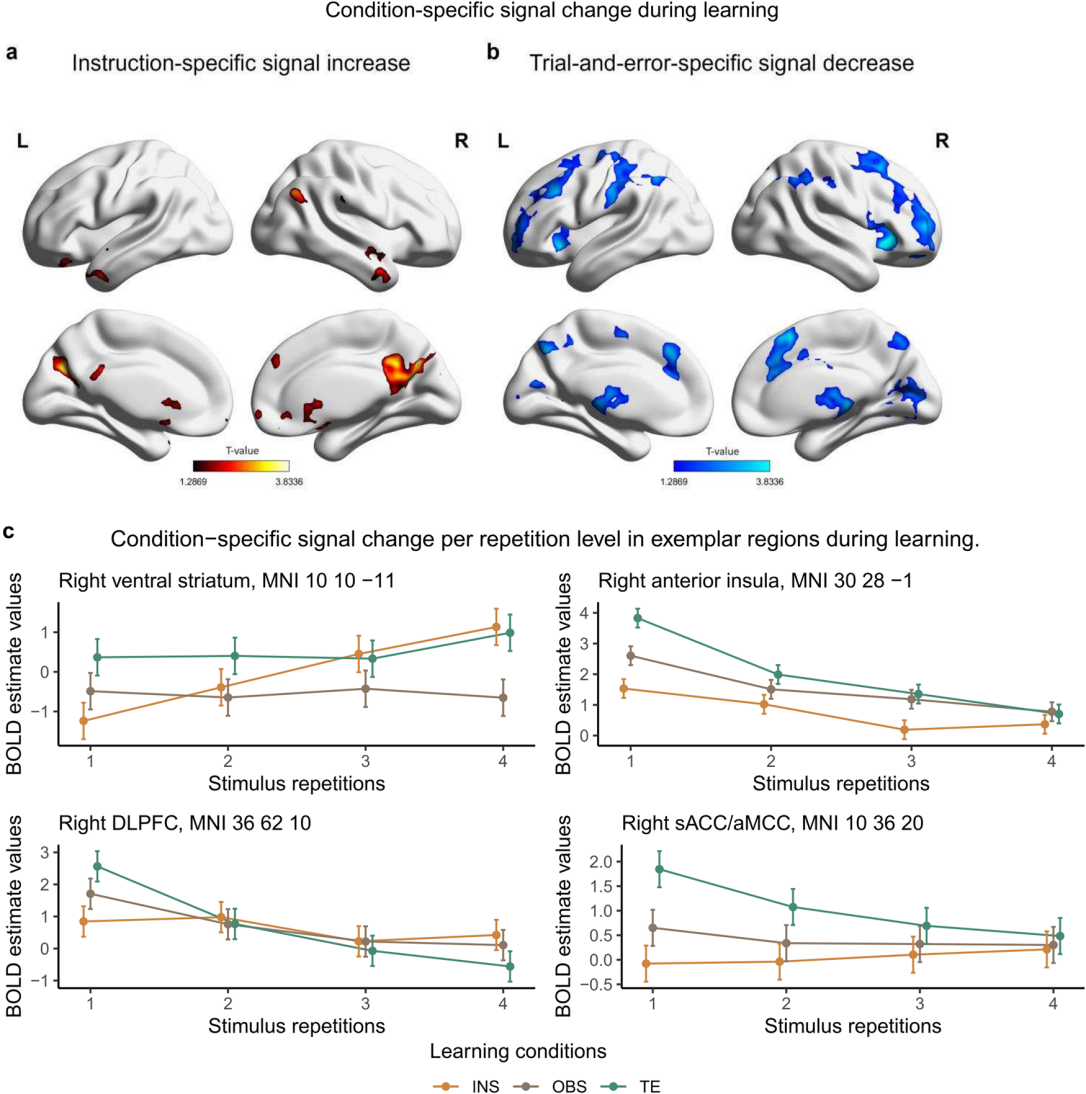
Condition-specific signal change during S-R learning. (a) Coronal and sagittal view of the brain for instruction-specific linear increase as compared to trial-and-error and observed trials. (b) Coronal and sagittal view of the brain for trial-and-error-specific linear decrease as compared to instructed and observed trials. (c) Exemplar regions for condition-specific signal change. INS = instruction, OBS = observation, TE = trial-and-error, DLPFC = Dorso-Lateral Prefrontal Cortex. T-maps (a, b) and MNI coordinates (c) result from the conjunction analyses (global null, FWE-cluster corrected, p < .05) that tested for condition-specific linear signal change during learning. BOLD estimate values come from repeated-measure ANOVA testing for the interaction learning condition * stimulus repetition. 90% confidence interval are plotted.

**Fig. 11.**
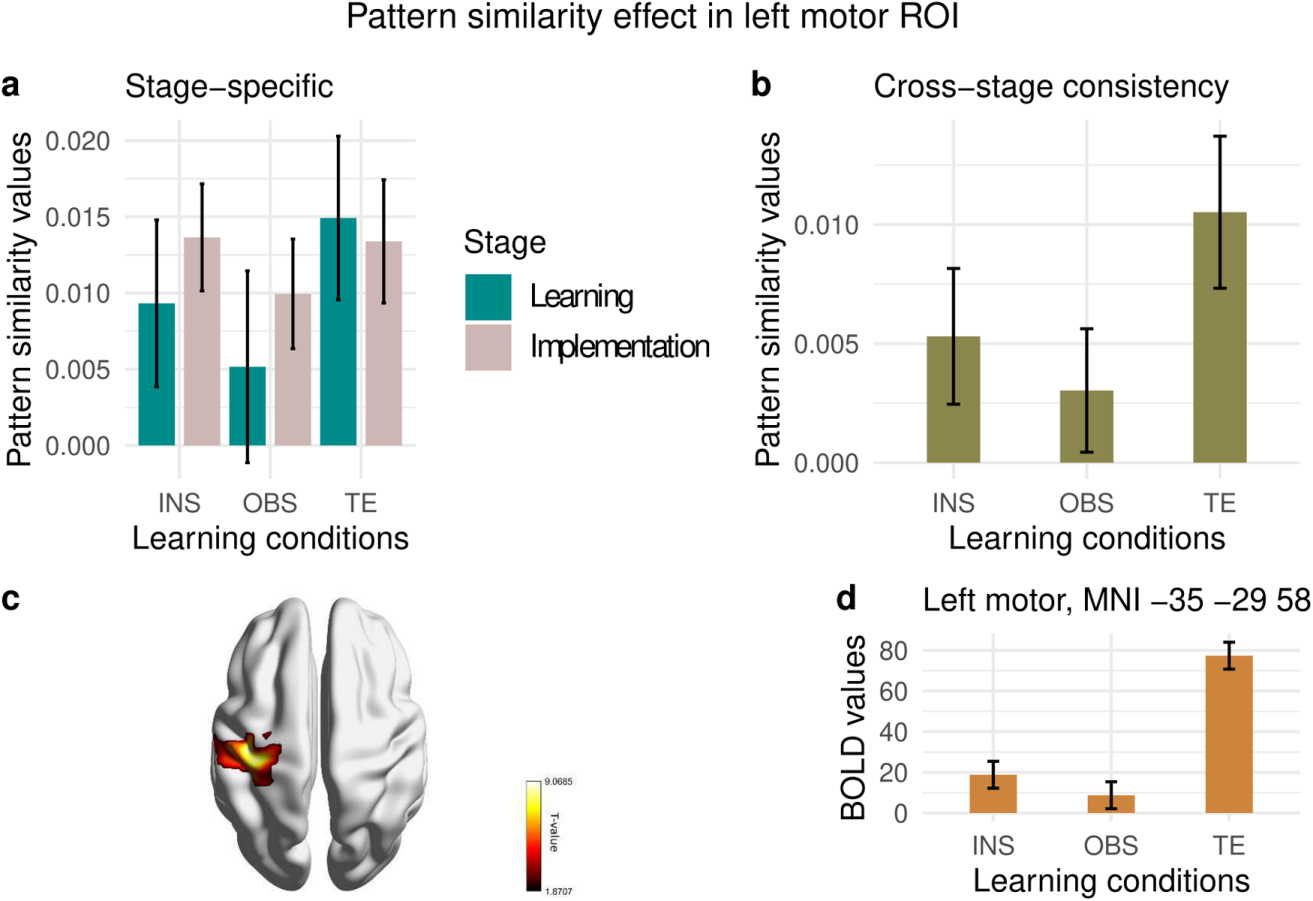
Pattern similarity effect in Left Motor ROI. 95% Confidence intervals are plotted. (a) Stage-specific pattern similarity analysis per each condition and stage. (b) Cross-stage consistency pattern similarity analysis per each condition. (c) Left motor cluster from conjunction analysis (FWE peak-level correction) for regions significantly active in trial-and-error more than instruction- and observation-based learning. (d) BOLD estimates plotted at the global maximum MNI coordinates [MNI -35 -29 58] of the left motor cluster as in (c). 90% confidence interval are plotted.

