# Supplementary material for: Initial learning in the brain: From rules to action
Source: Imaging Neurosci (Camb). 2024 Aug 20;2:imag-2-00274. doi: 10.1162/imag_a_00274 (PMC12327077; doi:10.1162/imag_a_00274)
Supplement: Supplementary Material [file imag_a_00274-supp.zip › imag_a_00274-supp.pdf]

Initial Learning in the Brain: From Rules to Action

Initial Learning in the Brain: From Rules to Action

Supplementary material

Preregistration

The study was initially preregistered on AsPredicted (https://aspredicted.org/9q9m6.pdf). Along the study, we made some changes that are described below.

Inclusion criteria. We initially planned to exclude subjects whose accuracy fell below 75% during implementation trials and 50% during learning trials for at least one stimulus in the trial-and-error condition. The rationale behind this was to optimize trial-and-error blocks before yoking them to the observation blocks between participants. However, this would have required analyzing subjects' performance during data acquisition to address potential dropouts and constantly have an available data set for the trial-and-error-to-observation block yoking procedure. Upon evaluating the feasibility of analyzing subjects' behavior during data acquisition, we realized that it posed challenges in terms of scanner booking times and participant scheduling. Consequently, we adopted a less stringent inclusion criterion, which was set at an 80% overall accuracy rate across conditions and blocks. The overall accuracy was calculated during the experiment by E-Prime itself and did not therefore require analysis behavior during data acquisition. Nonetheless, we conducted the pre-registered accuracy analysis at the end of the data acquisition phase. We identified seven participants who did not meet the pre-registered inclusion criteria for one to three stimuli in 1 out of 8 trial-and-error learning blocks (mean stimuli per block = 2.14, SD = 0.37). Subsequently, we examined the performance of these participants who were yoked in their observation-based learning blocks to those who did not perform well in the trial-and-error blocks. Surprisingly, all 'yoked' participants were able to successfully learn the S-R links.

In more detail, while participants from whom the response patterns were taken scored less than 50% and 75% hits in trial-and-error learning and implementation trials, respectively, the 'observation-yoked' participants met the 50-75 inclusion criterion. An example is provided in Table S1 for the observation condition (i.e., yoked participant) and Table S2 for the trial-and-error condition (i.e., source participant). Only one participant did not meet the 50-75 inclusion criterion in one observation block for 50% of the presented stimuli. However, in line with our approach of not excluding any other subjects or blocks analyzed within the scope of the 50-75 criterion, we decided to include this subject and block as well.

ROI-based MVPA contrasts. The planned stage-specific contrasts for repetition levels 1-2 vs. 3-4 and 5-6 vs. 7-8, as well as cross-stage consistency comparison of repetitions 3-4 vs. 5-6 were not implemented as pre-registered. Instead, the MVPA procedure was optimized to compute pairwise comparisons instead of mean values between repetitions of interest. This allowed for a more precise and reliable trial-by-trial analysis. For a more detailed description, refer to the MVPA analysis section.

Connectivity analysis. Given the length and complexity of the present paper, we have chosen to include the connectivity analysis in a separate paper.

MVPA sequence

MVPA can be biased due to systematic differences in model regressor correlations associated with the trial categories under investigation (e.g., Mumford et al. 2012). In the present rapid learning study, trial categories were individual stimulus-response (S-R) links and the aim was to identify multi-voxel activity patterns associated with them. To this end, we compared pattern similarities between repeated occurrences of the same S-R link (higher pattern similarity) with occurrences of different S-R links (lower pattern similarity).

Ruge et al. (2018) have shown that bias converges to zero if a random selection of all possible trial sequences is realized across different learning blocks and subjects.

Importantly, if sub-sections of the whole trial sequence are to be analyzed (e.g. in order to compare different learning stages), full randomization needs to be ensured for each such sub-section. For instance, if 4 S-R links are each repeated 8 times and trials are randomized across the whole 32 trial sequence, MVPA based on only the first (or last) 16 trials would be severely biased. Ruge et al. (2018) have demonstrated that a simplistic toy model using trial onset distances as a proxi for the resulting model regressor correlations is suited to already reveal how to properly design and evaluate trial sequences in order to avoid MVPA bias in subsequent full-fleshed simulations and real fMRI data analyses. Supplementary Fig. S1a is adopted from Ruge et al. (2018) and depicts the smallest possible toy model using 2 different stimuli each presented twice.

On average across all three possible trial sequence permutations, trial onset differences between same and different stimuli are identical. As confirmed by simulations, the analysis of real data, and by mathematical proof, this benign behavior generalizes to larger stimulus sets, greater number of repetitions, and is added random ITI jittering. As mentioned above, this holds as long as randomization applies to the entire trial sequence that is to be analyzed. Hence, if identity-specific pattern similiarities ought to be analyzed for distinct learning stages, randomization needs to be ensured stage-wise (the shortest possible learning stage would involve 2 repetitions of each individual S-R link).

Despite its merits, this trial sequencing scheme also comes with one huge limitation. Namely, bias-free pattern similarities can only be determine within each stage. It is not possible to compute similarities between stages! Yet, this was exactly one of the major aims of the present study. Fortunately, there is an alternative, maximally flexible sequencing scheme which accomplishes this goal while still ensuring unbiased results (Fig. S2). The trial sequencing scheme is depicted in supplementary Fig. S1b. The crucial trick is to only include trial sequences where a given stimulus is allowed to occur another time only after all stimuli have already occurred for the same number of times. Hence, in case of 4 different stimuli, an exemplary trial sequence would look be: S1 S4 S2 S3 / S3 S1 S2 S4 /

S2 S4 S1 S3 / S1 S2 S3 S4 / etc.

Moreover, in order to obtain zero bias, pattern similarity between different stimuli occurring within the same repetition have to be excluded from analysis.

Table S1

Example of single-subject performance in the observation-based learning condition when the source subject of the trial-and-error condition performed poorly in the learning stage.

Condition	Sub	Run	Block	TE Source Sub	Trial	Stimulus	Accuracy
OBS	13	4	7	8	3	1	0
OBS	13	4	7	8	8	1	0
OBS	13	4	7	8	10	1	0
OBS	13	4	7	8	16	1	1
OBS	13	4	7	8	19	1	1
OBS	13	4	7	8	21	1	1
OBS	13	4	7	8	27	1	1
OBS	13	4	7	8	31	1	1
OBS	13	4	7	8	1	4	0
OBS	13	4	7	8	6	4	0
OBS	13	4	7	8	11	4	0
OBS	13	4	7	8	15	4	1
OBS	13	4	7	8	18	4	1
OBS	13	4	7	8	24	4	1
OBS	13	4	7	8	25	4	1
OBS	13	4	7	8	29	4	1

OBS = Observation-based learning condition.

Sub = Subject

TE Source Sub = the subject from whom the trial-and-error learning response patterns were collected as part of the yoking procedure.

Table S2

Source subject's performance in the trial-and-error condition.

Condition	Sub	Run	Block	Trial	Stimulus	Accuracy
TE	8	4	8	3	1	0
TE	8	4	8	8	1	0
TE	8	4	8	10	1	0
TE	8	4	8	16	1	1
TE	8	4	8	20	1	0
TE	8	4	8	23	1	0
TE	8	4	8	27	1	0
TE	8	4	8	30	1	0
TE	8	4	8	1	4	0
TE	8	4	8	6	4	0
TE	8	4	8	11	4	0
TE	8	4	8	15	4	1
TE	8	4	8	19	4	0
TE	8	4	8	24	4	0
TE	8	4	8	26	4	0
TE	8	4	8	31	4	0

TE = Trial-and-Error learning condition.

Sub = Subject

Table S3

Descriptive statistics of trial-and-error learning trials per each stimulus repetition.

Stimulus repetition	RT_mean	RT_sd	Acc_mean	Acc_sd
1	660.761	213.138	37.5	48.4
2	783.707	228.393	70.0	45.8
3	755.612	222.321	87.5	33.1
4	733.324	200.326	94.0	23.8

Accuracy (%) and RTs (ms, only correct trials).

Table S4

Descriptive statistics of implementation trials per stimulus repetition and condition.

Learning condition	Stimulus repetition	RT_mean	RT_sd	Acc_mean	Acc_sd
INS	1	630.967	170.740	97.8	14.8
OBS	1	660.381	200.548	95.7	20.2
TE	1	635.531	184.833	97.5	15.7
INS	2	641.473	163.644	98.3	13.0
OBS	2	661.688	185.705	96.3	18.9
TE	2	651.812	172.491	97.2	16.4
INS	3	645.177	166.646	97.7	14.9
OBS	3	658.898	173.921	97.1	16.8
TE	3	648.885	162.988	97.7	15.0
INS	4	646.875	158.529	97.8	14.6
OBS	4	656.258	170.140	97.0	17.2
TE	4	650.902	163.679	97.1	16.9

Accuracy (%) and RTs (ms, only correct trials). INS = instruction, OBS = observation, TE = trial-and-error.

Table S5

Descriptive statistics of implementation trials per stimulus repetition across conditions.

Stimulus repetition	RT_mean	RT_sd	Acc_mean	Acc_sd
1	637.566	201.979	97.0	17.1
2	648.458	185.877	97.3	16.3
3	648.660	179.140	97.5	15.6
4	648.094	174.844	97.3	16.3

Accuracy (%) and RTs (ms, only correct trials).

Table S6

Descriptive statistics of implementation trials per condition across stimuulus repetitions.

Learning condition	RT_mean	RT_sd	Acc_mean	Acc_sd
INS	636.822	175.466	97.9	14.3
OBS	654.030	199.237	96.5	18.3
TE	646.231	181.483	97.4	16.0

Accuracy (%) and RTs (ms, only correct trials). INS = instruction, OBS = observation, TE = trial-and-error.

Table S7

False alarms in instructed learning trials.

Subject	Block	Trial/block	Acc	RT (ms)	FAs (n)	FAs (%)
14	3	1	1	841	1	0.78
21	10	1	1	875	1	0.78
26	5	5	1	787	1	0.78
28	6	5	1	1236	1	0.78
35	1	2	1	584	1	0.78
37	2	5	1	644	1	0.78
39	2	1	0	847	1	0.78
48	11	12	1	1135	2	1.56
	11	13	1	683		
52	6	7	1	555	1	0.78
56	1	4	1	1114	3	2.34
	9	10	1	597		
	11	1	1	1231		
59	13	1	1	1431	1	0.78
61	23	9	1	689	1	0.78
79	8	5	1	772	1	0.78

The table displays false alarms (FAs) committed by subjects during instructed learning trials, categorizing them based on accuracy (Acc = 0 for incorrect, Acc = 1 for correct). Details include block, trial within the block, and reaction time (RT), the latter measured from the onset of the stimulus. Total number and percentage of false alarms for each subject who committed FAs across the whole experiment are also displayed. The mean FAs across the experiment and the whole sample equal 0.2% in instructed learning trials.

Table S8

False alarms in observed learning trials.

Subject	Block	Trial/block	Acc	RT (ms)	ObsResp.OnsetTime	FAs (n)	FAs (%)
14	14	7	0	712	767	1	0.78
20	15	12	1	826	976	1	0.78
21	21	1	0	759	1076	1	0.78
24	21	15	1	565	901	1	0.78
25	14	1	0	640	816	1	0.78
33	24	14	1	797	1109	1	0.78
35	12	3	0	469	597	1	0.78
37	15	9	1	874	1074	1	0.78
47	15	11	1	694	898	1	0.78
48	12	13	1	606	1360	1	0.78
52	4	7	1	692	776	1	0.78
54	10	12	1	680	857	1	0.78
64	7	13	1	916	1055	1	0.78
70	1	13	0	691	909	1	0.78
76	11	2	0	624	906	1	0.78
80	16	14	1	543	1075	1	0.78

The table displays false alarms (FAs) committed by subjects during observed learning trials, categorizing them based on accuracy (Acc = 0 for incorrect, Acc = 1 for correct). Details include block, trial within the block, reaction time (RT), and observed response onset time, both measured from the onset of the stimulus. Total number and percentage of false alarms for each subject who committed FAs across the whole experiment are also displayed. The mean FAs across the experiment and the whole sample equal 0.2% in observed learning trials.

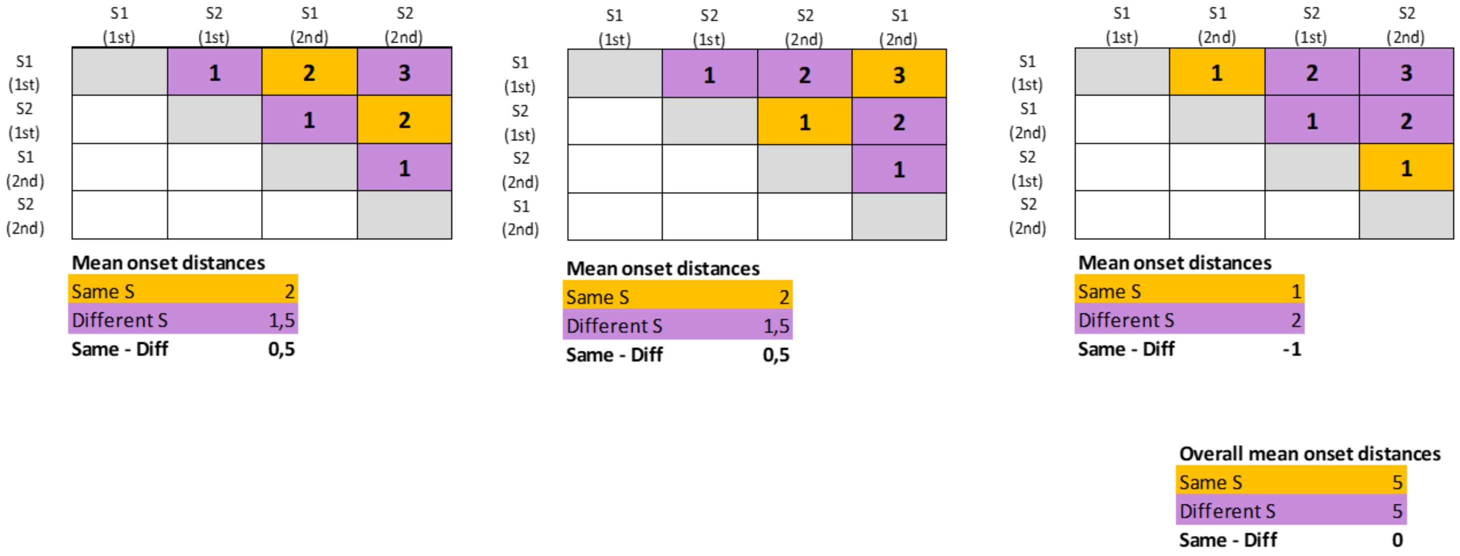

(a) Original MVPA scheme with toy model using 2 stimuli x 2 repetitions per stimulus as in Ruge et al. (2018)

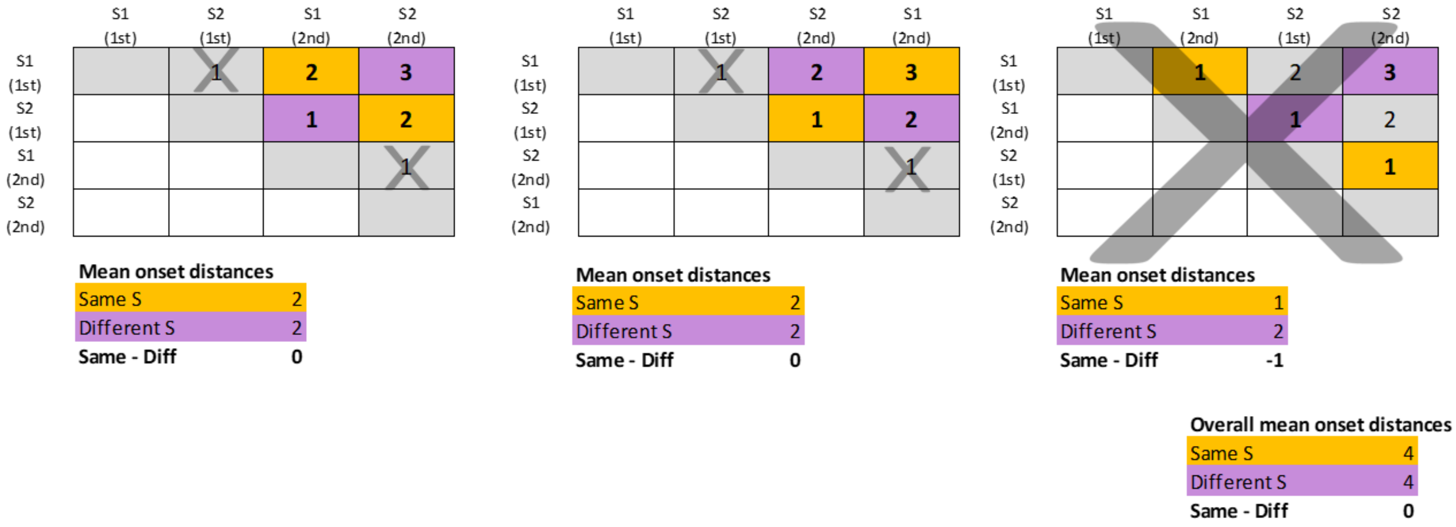

(b) Alternative MVPA scheme with toy model using 2 stimuli x 2 repetitions per stimulus as in the present paper.

Figure S1. a) Original MVPA scheme as in Ruge et al. (2018). b) Alternative MVPA scheme as in the present paper. Both schemes are generated with a toy model which uses trial onset distances using 2 stimuli x 2 repetitions per stimulus as a proxi for bias in MVPA. In each table, "same S" refers to the same stimulus and "different S" to different stimuli, whereas 1st and 2nd refer to the stimulus repetition level. Trial onset differences between same and different stimuli are computed per model and across models, leading to the mvpa bias proxi value.

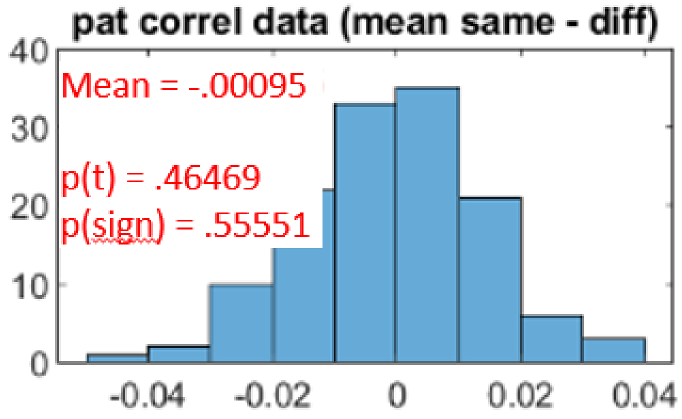

Figure S2. Simulation results from the MVPA sequence adopted in the present paper. The barplot demonstrate unbiased identity-specific pattern similarity estimates (see Ruge et al. 2018 for details on simulation methods). Mean identity-specific pattern similarity (i.e., mean pattern similarities for same stimuli vs. different stimuli) is not significantly different from zero indicating unbiased results. p(t): H0 probability under t test: p(sign): H0 probability under sign test.

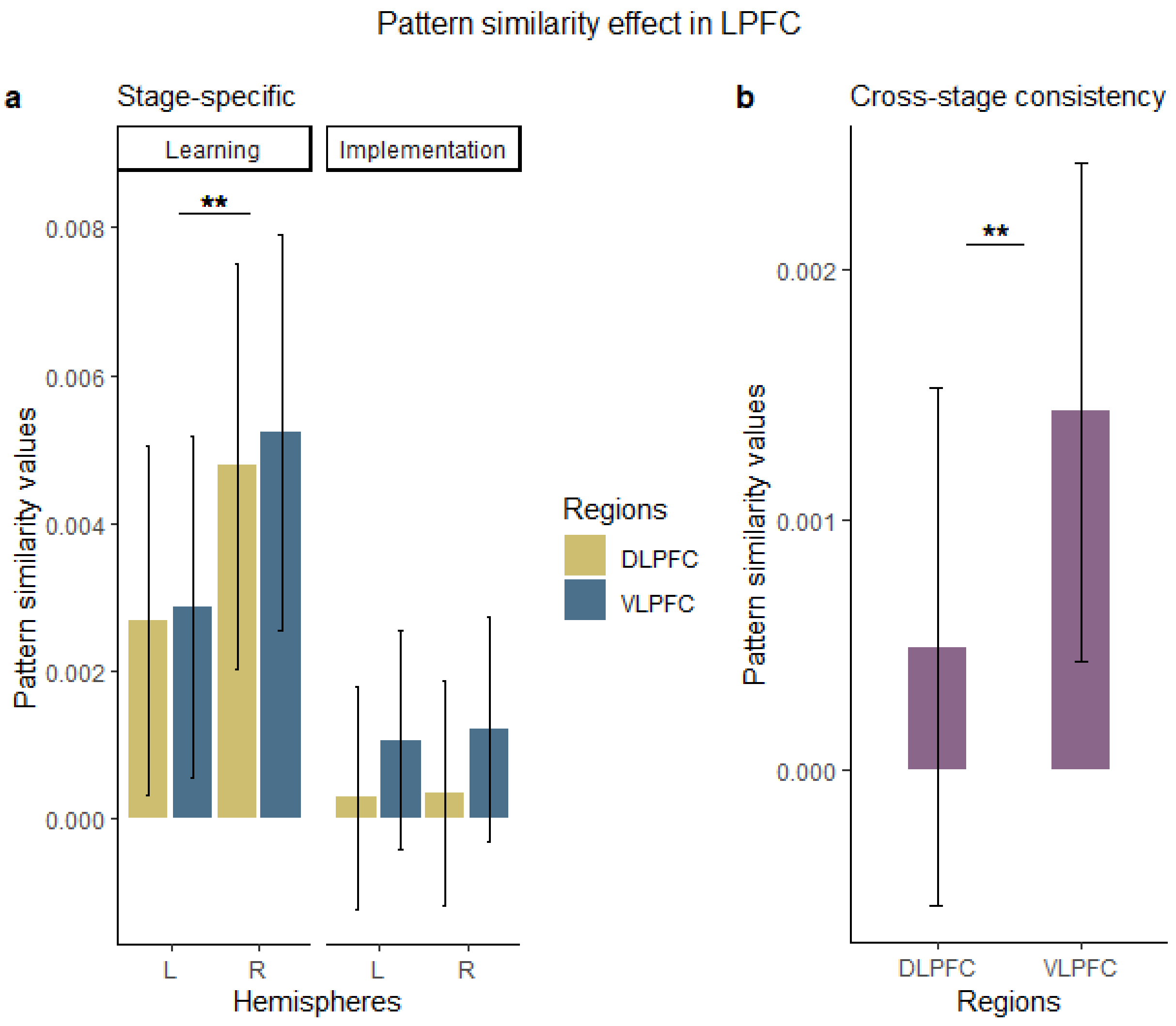

Figure S3. Pattern similarity effect in ventrolateral VLPFC and dorsolateral DLPFC prefrontal cortex. 95% Confidence intervals are plotted. Figure depicts significant effects.

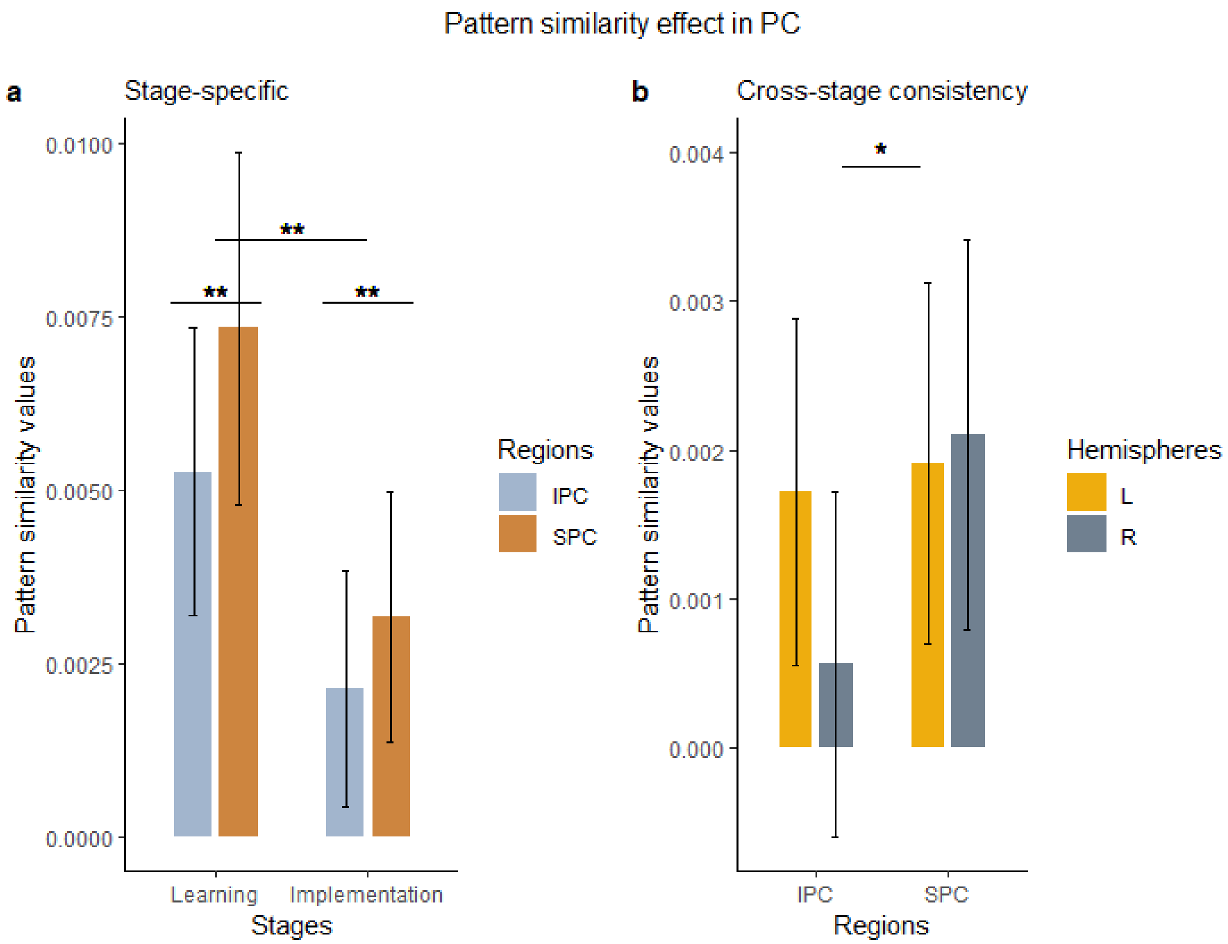

Figure S4. Pattern similarity effect in superior SP and inferior parietal IP cortex. 95% Confidence intervals are plotted. Figure depicts significant effects.

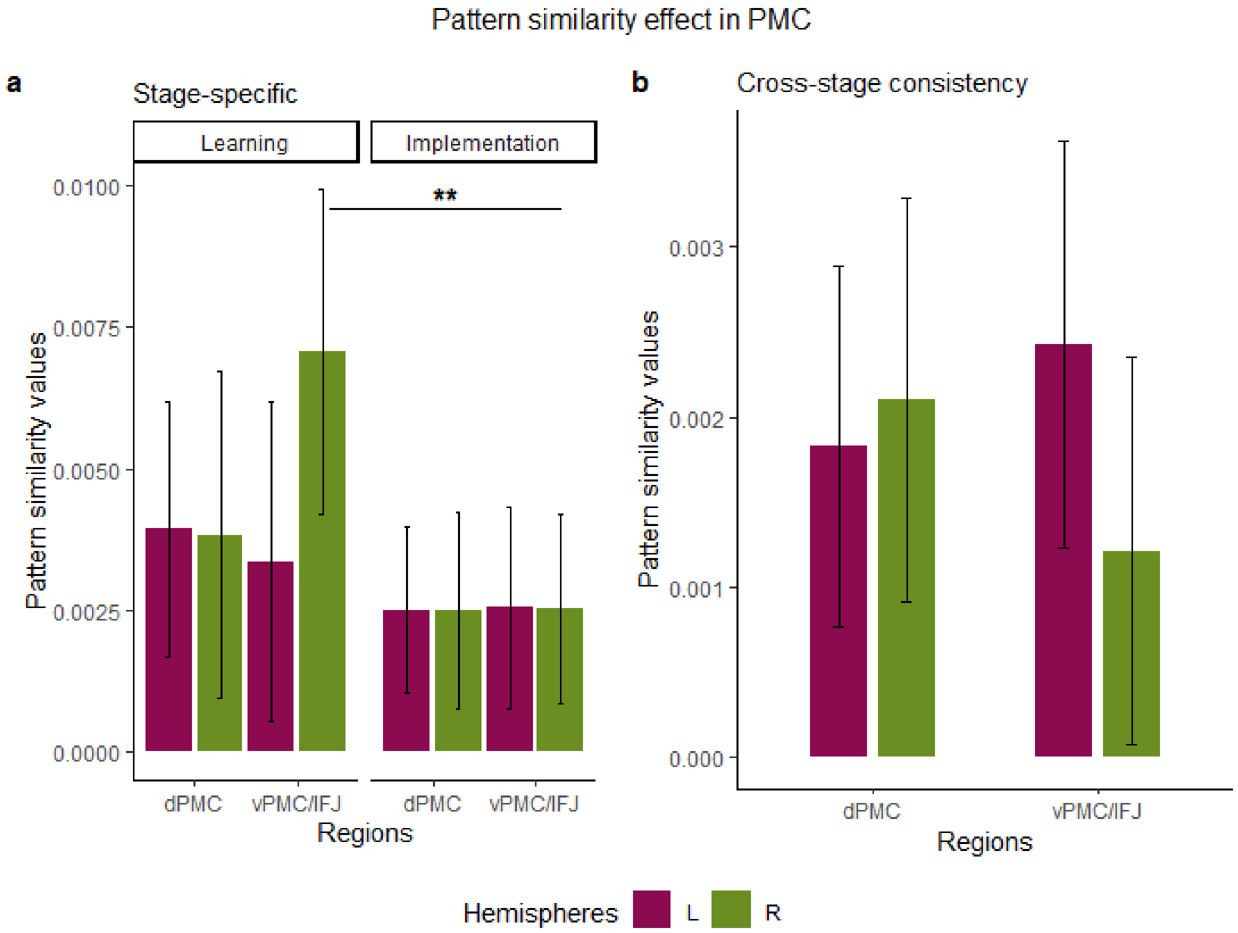

Figure S5. Pattern similarity effect in Premotor Cortex, PMC. dPMC = Dorsal Premotor Cortex; vPMC/IFJ = Ventral Premotor Cortex/Inferior Frontal Junction. 95% Confidence intervals are plotted. Figure depicts significant effects.
